# Synergistic Sorption of Gallium(III) and Scandium(III) Ions by the Interpolymer System of Poly(Acrylic Acid)/Poly(4-vinylpyr-idine) Hydrogels: Kinetics, Selectivity, and Mechanism

**DOI:** 10.3390/molecules31183324

**Published:** 2026-09-19

**Authors:** Bakytgul Totkhuskyzy, Talkybek Jumadilov, Khuangul Khimersen, Arailym Akhmetova, Józef Haponiuk, Juozas Grazulevicius

**Affiliations:** 1 Bekturov Institute of Chemical Sciences, 106 Valikhanov Str., Almaty 050010, Kazakhstan; jumadilov@mail.ru; 2 Department of Biotechnology and General Chemical Technology, School of Pharmacy, Asfendiyarov Kazakh National Medical University, 94 Tole bi Str., Almaty 050012, Kazakhstan; 3 Department of Chemistry, Institute of Natural Sciences and Geography, Abai Kazakh National Pedagogical University, 13 Dostyk Ave., Almaty 050010, Kazakhstan; 4 School of Chemical Engineering, Kazakh-British Technical University, 59 Tole bi Str., Almaty 050000, Kazakhstan; 5 Department of Polymer Technology, Gdansk University of Technology, 11/12 Gabriela Narutowicza, 80-233 Gdansk, Poland; jozef.haponiuk@pg.edu.pl; 6 Department of Polymer Chemistry and Technology, Kaunas University of Technology, 73 K. Donelaičio Street, 44249 Kaunas, Lithuania

**Keywords:** intergel hydrogels, poly(acrylic acid), poly(4-vinylpyridine), gallium(III) sorption, scandium(III) sorption, selective separation, hydrometallurgy, Langmuir isotherm

## Abstract

Selective recovery of critical metals such as gallium and scandium from dilute, multicomponent process streams remains challenging due to their low concentrations and complex matrices. Here we investigate a remotely interacting intergel system based on crosslinked poly(acrylic acid) (hPAA) and poly(4-vinylpyridine) (hP4VP) hydrogels across seven mass ratios (6:0–0:6) for sorption of Ga(III) and Sc(III) from binary sulfate solutions. Inductively coupled plasma optical emission spectroscopy shows that mutual activation of the weak polyacid/polybase pair markedly enhances metal uptake, with maximum sorption degrees of 73.17% for Ga(III) at hPAA:hP4VP = 3:3 and 56.00% for Sc(III) at 2:4, corresponding to distribution coefficients up to 2726.7 mL g^−1^ and Ga/Sc selectivity factors β up to 6.41. Sorption is well described by Langmuir-type isotherms, and desorption with 2 wt.% HNO_3_ reveals an inverse sorption–release relationship, indicating stronger binding at compositions of maximal mutual activation. FTIR spectroscopy evidences inner-sphere complexation through bidentate carboxylate coordination in hPAA and M←N dative bonding in hP4VP, while TGA/DSC shows that metal-loaded hydrogels retain their structural integrity up to approximately 320–340 °C, with the onset of significant decomposition occurring at ~350 °C for hP4VP-containing samples. The composition-dependent magnitude of Ga(III)-over-Sc(III) preferential uptake—with selectivity coefficients β = K_d_(Ga)/K_d_(Sc) remaining greater than 1 across all seven compositions studied—is rationalized using Hard–Soft Acid–Base considerations and demonstrates that hPAA:hP4VP intergels constitute a composition-tunable platform for enhancing Ga(III) recovery and partial Ga/Sc separation from simplified sulfate media, motivating future validation in real hydrometallurgical liquors and in the presence of competing multivalent cations.

## 1. Introduction

Gallium and scandium are strategic dispersed elements that play a key role in advanced technologies, including microelectronics, optoelectronics, high-efficiency lighting, aerospace alloys and functional ceramics. Gallium is an essential component of compound semiconductors such as GaAs and GaN used in light-emitting diodes, high-frequency and power electronics, and third-generation photovoltaic devices [1,2]. Scandium is a highly effective modifier of aluminum alloys, providing low density, high strength and excellent corrosion resistance that are attractive for aerospace, automotive and energy applications. Despite their technological importance, both elements are classified as critical due to their low natural abundance, dispersed occurrence and the absence of dedicated large-scale ore deposits [3]. Gallium is mainly recovered as a by-product of bauxite and zinc processing [4], while scandium is obtained from red mud, coal combustion products and rare earth ores [5]. The low concentrations of Ga(III) and Sc(III) in process streams, together with complex multicomponent matrices, make their efficient and selective recovery a challenging task.

At present, industrial recovery of gallium and scandium relies on combinations of precipitation, solvent extraction, cementation and ion exchange [6,7,8,9]. These methods are often multi-stage, energy-intensive and sensitive to solution composition, and they may suffer from limited selectivity, substantial reagent consumption and difficulties in handling complex wastes such as red mud. The development of sorbents capable of selectively binding Ga(III) and Sc(III) from dilute, strongly alkaline or acidic process liquors is therefore of considerable interest. In this context, polymeric hydrogels and ion-exchange materials based on poly(acrylic acid) (hPAA) and nitrogen-containing polymers have attracted attention due to their high hydrophilicity, tunable charge density and the possibility of forming interpolymer complexes. Interpolymer systems [10] combining weak polyacids and polybases can exhibit cooperative binding and enhanced sorption performance compared to their single-component analogues, particularly when the ratio and spatial arrangement of the components are controlled.

Interpolymer systems formed by poly(acrylic acid) and poly(4-vinylpyridine) represent a promising class of materials [11,12,13,14,15], in which carboxylic and pyridine groups participate in acid–base interactions and metal coordination. However, the potential of PAA/P4VP-based hydrogels for the recovery and separation of Ga(III) and Sc(III) from aqueous solutions has not yet been systematically explored. In particular, the influence of the PAA:P4VP ratio and the spatial organization of the components on sorption capacity, kinetics and selectivity remains insufficiently understood.

Beyond conventional ion-exchange resins and solvent extraction systems, several advanced sorbents have been reported for Ga and Sc recovery, including functionalized chitosan–silica hybrids [16,17,18], phosphorylated polymers [19,20,21], and tailored organophosphorus extractants [22]. More broadly, the design of compositionally tuned functional materials remains an active area of research across separation science, catalysis, and composite materials, since relatively small changes in composition, additive identity, and local chemical environment can substantially affect physicochemical behavior and performance. In polymer-based sorbents [23,24,25], these composition–structure relationships are especially important because the ratio, functionality, and spatial arrangement of the components determine the accessibility of active sites, the swelling behavior of the network, and the strength of metal–ligand interactions. In this context, intergel systems based on commercial or readily accessible hydrogels offer a simple physical design in which mutual activation via remote acid–base interaction can be exploited as an additional control parameter for sorption performance, as demonstrated previously for Au(III), Y(III), and rare earth ions. However, Ga(III)/Sc(III) systems have not yet been investigated in this framework, despite their high strategic importance and challenging co-occurrence in hydrometallurgical process liquors.

In this work we test the hypothesis that mutual activation between hPAA and hP4VP under remote contact conditions generates composition-dependent, HSAB-governed binding environments that can be tuned to preferentially sorb Ga(III) or Sc(III) from binary sulfate solutions. We investigate hPAA:hP4VP intergel systems prepared at seven mass ratios under remote contact, quantify sorption kinetics and distribution coefficients for Ga(III) and Sc(III) using ICP-OES, and evaluate desorption with dilute nitric acid. FTIR spectroscopy and TGA/DSC are employed to elucidate the coordination mechanism and thermal stability of metal-loaded hydrogels. By combining these approaches, we aim to identify optimal compositions that maximize sorption efficiency and compositional selectivity, and to assess the potential of hPAA:hP4VP intergels for integration into sorption-based flowsheets for the recovery and partial separation of Ga(III) and Sc(III) from hydrometallurgical process solutions.

## 2. Results

### 2.1. Sorption and Desorption of Gallium from a Mixed Solution

Sorption of Ga^3+^ and Sc^3+^ ions was carried out in the intergel system hPAA:hP4VP at varying mass ratios of the polymer hydrogels (6:0, 5:1, 4:2, 3:3, 2:4, 1:5, and 0:6) in model mixed solutions of gallium(III) sulfate and scandium(III) sulfate, with initial concentrations of Ga^3+^ and Sc^3+^ of 30 mg/L each. Kinetic measurements were performed at contact time intervals of 0.5, 1, 2, 4, 6, 24, and 48 h. The equilibrium residual concentrations of Ga^3+^ and Sc^3+^ ions remaining in solution after sorption by the respective intergel systems are presented in Table 1 and Table 2.

**Figure 1:**
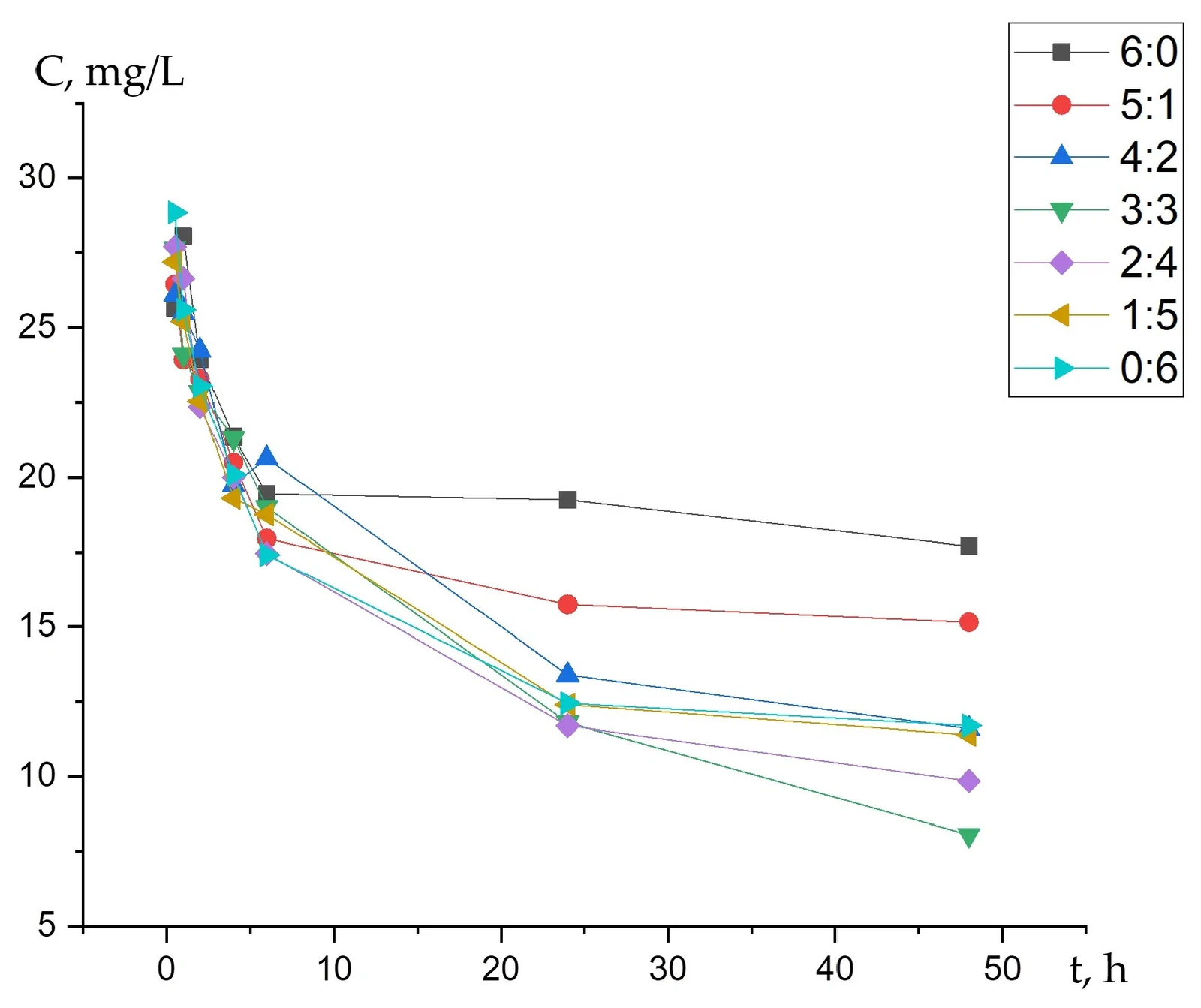
Dependence of the residual concentration of gallium (III) ions in solution after sorption on the mass ratio of polymers in the hPAA:hP4VP interpolymer system and on contact time.

Based on ICP-AES data, a plot of the residual concentration of gallium(III) ions in solution as a function of contact time and polymer mass ratio in the intergel system hPAA:hP4VP was constructed (Figure 1). The obtained curves clearly illustrate the sorption kinetics of Ga^3+^ ions across the full range of sorbent compositions studied (hPAA:hP4VP = 6:0, 5:1, 4:2, 3:3, 2:4, 1:5, and 0:6), demonstrating the dynamic character of ion uptake and enabling direct comparison of the sorption efficiency at each polymer ratio over the entire contact time interval from 0.5 to 48 h.

As seen from Figure 1, a progressive decrease in the residual Ga^3+^ concentration is observed with increasing contact time for all polymer compositions, with the most pronounced reduction achieved at the ratio corresponding to maximum mutual activation of the hPAA and hP4VP networks. The system reaches a near-equilibrium state within 48 h, after which no significant further decrease in residual concentration is observed.

The degree of sorption of Ga^3+^ and Sc^3+^ ions was calculated from the residual concentration data obtained by ICP-AES using Equation (3). The resulting values for gallium(III) are summarized in Table 2.

The concentration of gallium(III) ions in the desorbing solutions was determined by ICP-OES. Desorption was carried out using the same intergel systems (hPAA:hP4VP mass ratios of 6:0, 5:1, 4:2, 3:3, 2:4, 1:5, and 0:6) following completion of the sorption process. Unlike the kinetic sorption studies—in which aliquots were sampled at multiple time intervals—desorption measurements involved the collection of a single aliquot from each system after 48 h of contact with a 2 wt.% aqueous nitric acid (HNO_3_) solution. The degree of desorption (R, %) was calculated according to Equation (4).

### 2.2. Desorption of Ga^3+^ Ions

Following successful sorption, the reverse process of gallium(III) desorption was investigated to assess the reusability of the hPAA-hP4VP interpolymer system and to gain further insight into the nature of the polymer–metal ion interactions established during uptake. Desorption experiments were carried out using 2 wt.% HNO_3_ as the eluent, with contact times of up to 48 h, and were performed across the full range of hPAA:hP4VP mass ratios (6:0 to 0:6) studied previously in the sorption stage. Acidification of the medium was expected to protonate the carboxylate and pyridine functional groups of hPAA and hP4VP, respectively, thereby weakening the coordination bonds formed with Ga^3+^ during sorption and promoting its release back into the aqueous phase. The resulting residual Ga^3+^ concentrations and corresponding desorption efficiencies were compared across compositions to determine how the polymer ratio governs the strength and reversibility of metal-ion binding within the interpolymer network.

As evident from Table 3, the highest concentration of gallium(III) ions in the aqueous phase following the desorption process was recorded at hPAA:hP4VP ratios of 4:2 (67% hPAA: 33% hP4VP) and 3:3 (50% hPAA: 50% hP4VP) after 48 h of contact with the desorbent solution. This finding correlates with the mutual activation effect characteristic of intergel systems at intermediate polymer ratios, wherein the enhanced ionization of both hPAA and hP4VP networks during the sorption stage leads to stronger initial metal binding, and consequently to a more pronounced release of Ga^3+^ ions upon acidification. The predominance of hPAA in the 4:2 composition suggests that carboxylate functional groups play a central role in gallium coordination, with protonation of –COO^−^ groups by HNO_3_ driving the displacement of Ga^3+^ ions back into solution.

As shown in Table 4, the desorption degree of Ga^3+^ ions after 48 h of contact with 2 wt.% HNO_3_ ranged from 55.46% to 75.82% across all studied hPAA:hP4VP compositions. The highest desorption efficiency was observed for the interpolymer system (4:2 ratio), reaching 75.82%, followed by the 5:1 (71.04%) and 3:3 (66.97%) compositions. The lowest desorption degree was recorded at the 0:6 ratio (55.46%), suggesting that equimolar polymer compositions—which are known to exhibit the strongest mutual activation and consequently the deepest Ga^3+^ binding—are the most resistant to acid-driven desorption. These results indicate that while 2 wt.% HNO_3_ is effective as a desorbent across all compositions, the degree of metal release is inversely related to the strength of polymer–ion interaction established during the sorption stage.

The inverse trend between sorption and desorption degrees for Sc^3+^ contrasts with the behavior of Ga^3+^ (Table 4), where the highest desorption was observed for the individual hP4VP hydrogel (0:6, 55.46%). This divergence suggests that the intergel system exhibits differential selectivity toward Ga^3+^ and Sc^3+^ not only during sorption but also during the regeneration stage, which has important implications for the potential separation of these two metals using hPAA:hP4VP systems.

### 2.3. Sorption and Desorption of Scandium from a Mixed Solution

**Figure 2:**
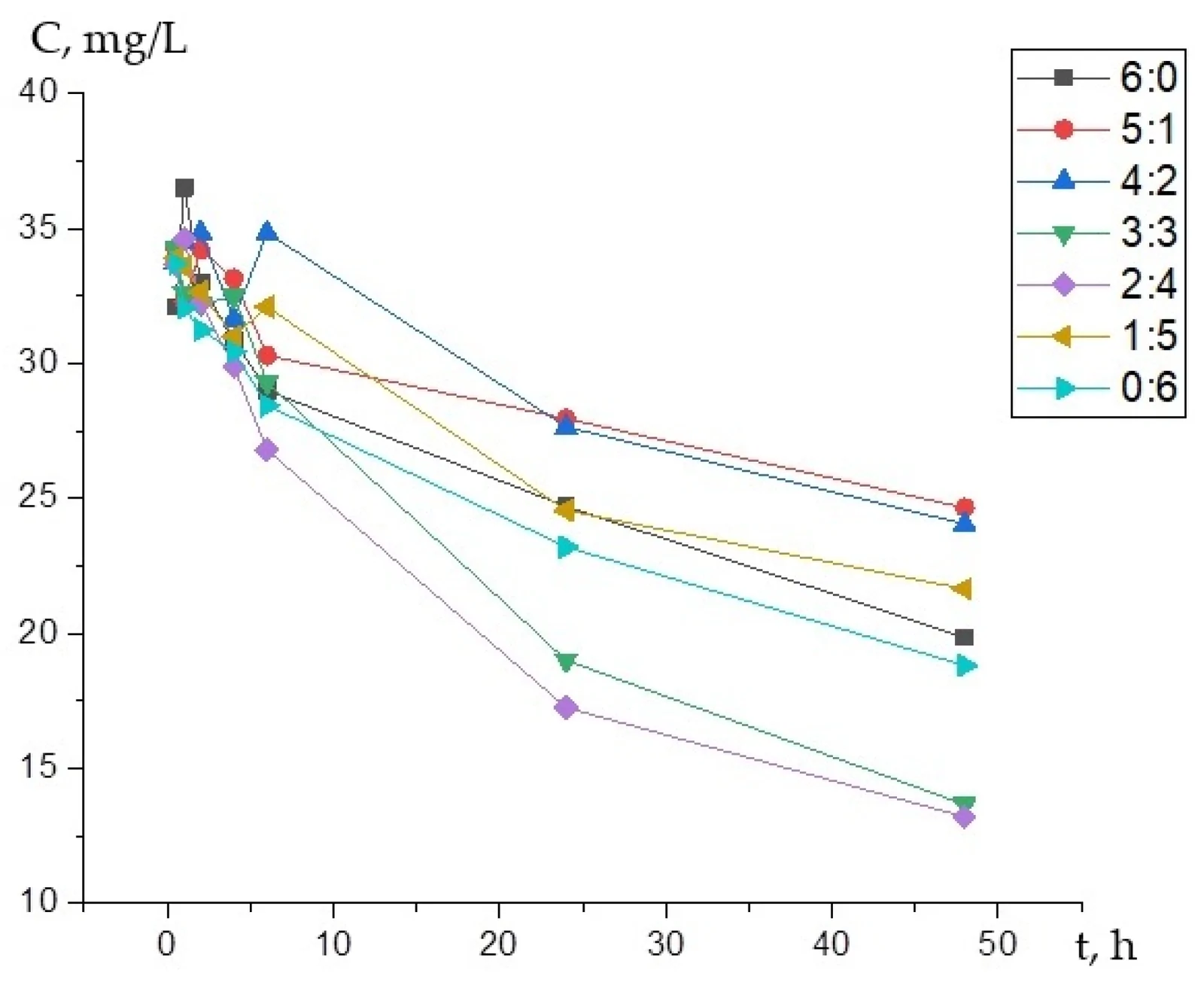
Residual concentration of scandium(III) ions in solution after sorption as a function of polymer mass ratio in the intergel system hPAA:hP4VP and contact time.

The following data present the results of inductively coupled plasma optical emission spectrometry (ICP-OES) analysis of scandium ion concentrations extracted from the mixed metal solution. The residual concentrations of Sc^3+^ ions remaining in solution after the sorption process by the hPAA:hP4VP intergel system at all studied polymer ratios and contact times are summarized in Table 5, and the corresponding kinetic profiles are illustrated graphically in Figure 2.

As presented in Table 5, the residual concentration of Sc3+ ions in solution at early contact times (0.5–2 h) slightly exceeds the initial concentration of 30 mg/L across nearly all polymer compositions. This apparent superequilibrium concentration could, in principle, arise from several distinct causes: (i) displacement of residual pore- or surface-bound solution from the hydrogel network during the initial rapid swelling phase, which would transiently increase the ion concentration in the bulk solution sampled for analysis; (ii) leaching of trace Sc-containing or interfering species from the polymer or crosslinker itself; or (iii) analytical variability associated with ICP-OES measurement at low contact times when the system has not yet reached steady state.

To test these hypotheses, a control experiment was conducted in which pristine hP4VP and hPAA hydrogels were separately equilibrated in metal-free 0.05 M Na_2_SO_4_ solution (matched ionic strength, pH 2.5 ± 0.1) for 0.5–2 h under stirring and temperature conditions identical to the sorption experiments; the resulting solutions were analyzed by ICP-OES for Sc traces. No detectable Sc was found in either blank, ruling out leaching from the polymer or crosslinker (hypothesis ii) as a contributing cause. Triplicate measurements of the sorption experiment at the 0.5 h and 1 h time points showed a standard deviation of ≤5% of the mean residual Sc^3+^ concentration, consistent with the instrumental uncertainty stated in Section 3.1. Because no Sc leaching was detected and the observed variability at these time points falls within the stated measurement uncertainty, we attribute the apparent superequilibrium concentrations primarily to swelling-induced displacement of pore/surface-bound solution (hypothesis i) combined with normal ICP-OES measurement variability at low contact times (hypothesis iii), rather than to trace contamination or leaching.

At 48 h of contact, the highest degrees of scandium(III) sorption were achieved at hPAA:hP4VP ratios of 2:4 (56.00%) and 3:3 (54.50%), with residual concentrations of 13.20 mg/L and 13.65 mg/L, respectively. These results indicate that compositions with a predominance of hP4VP—or equimolar polymer content—favor Sc^3+^ uptake, likely due to the role of pyridine nitrogen groups of hP4VP in coordinating Sc^3+^ ions. By contrast, individual hydrogels (6:0 and 0:6) and compositions with higher hPAA content showed comparatively lower sorption efficiency (19.85 and 18.80 mg/L residual, respectively), confirming that the mutual activation effect in the intergel system at intermediate ratios enhances Sc^3+^ sorption beyond the capacity of either individual polymer.

Figure 2 illustrates the kinetic profiles of residual Sc^3+^ concentration across all studied hPAA:hP4VP compositions (6:0, 5:1, 4:2, 3:3, 2:4, 1:5, and 0:6) over a contact time range of 0.5 to 48 h. As seen from the figure, all compositions exhibit a gradual decline in residual Sc^3+^ concentration with increasing contact time, with the most pronounced decrease observed at ratios 2:4 and 3:3, reaching residual concentrations of 13.20 mg/L and 13.65 mg/L, respectively, after 48 h. The curves corresponding to compositions with higher hP4VP content (2:4 and 3:3) lie consistently below those of hPAA-dominant compositions at extended contact times, visually confirming the superior affinity of Sc^3+^ ions toward polymer systems enriched in pyridine nitrogen functional groups. The relatively flat profiles observed at early time points (0.5–2 h) reflect the initial swelling and conformational reorganization of the hydrogel networks prior to the onset of effective ion uptake, consistent with the behavior previously reported for intergel systems under remote interaction conditions.

As seen in Figure 2, the minimum residual concentrations of scandium(III) ions in solution after sorption are achieved in intergel systems with hPAA:hP4VP mass ratios of 3:3 (50% hPAA: 50% hP4VP) and 2:4 (33% hPAA: 67% hP4VP) at a contact time of 48 h.

Table 6 demonstrates the calculated degrees of Sc^3+^ sorption (η) derived from the equilibrium concentration data obtained by ICP-OES for the mixed gallium–scandium solution.

As shown in Table 6, the maximum degrees of Sc^3+^ sorption at 48 h were attained at ratios 2:4 (56.00%) and 3:3 (58.57%), confirming that compositions with equal or hP4VP-dominant polymer content provide the most effective uptake of scandium ions from the mixed solution. Positive sorption degrees become consistently established from 4–6 h of contact onward across all compositions, with values increasing substantially between 6 and 24 h, indicating that the principal sorption occurs during this interval. The negative sorption degree values observed at early contact times (0.5–2 h) are characteristic of intergel systems and reflect the initial swelling-induced displacement of solution volume rather than true metal release.

### 2.4. Desorption of Sc^3+^ Ions

The concentration of scandium(III) ions in the desorbing solutions was determined by ICP-OES. Desorption was performed using the same intergel systems (hPAA:hP4VP ratios of 6:0, 5:1, 4:2, 3:3, 2:4, 1:5, and 0:6) following completion of the sorption process. Unlike the kinetic sorption measurements—in which aliquots were collected at multiple time intervals—desorption analysis involved collection of a single aliquot from each system after 48 h of contact with a 2 wt.% HNO_3_ solution. The resulting residual Sc^3+^ concentrations and desorption degrees are presented in Table 7 and Table 8.

As evident from Table 7, the highest concentrations of scandium(III) ions in the aqueous phase following the desorption process were observed at hPAA:hP4VP ratios of 2:4 (33% hPAA: 67% hP4VP) and 3:3 (50% hPAA: 50% hP4VP), reaching 22.00 mg/L and 21.40 mg/L, respectively, after 48 h of remote interaction with 2 wt.% HNO_3_.

This finding is consistent with the sorption results presented in Table 7, where these same compositions demonstrated the highest Sc^3+^ uptake—the greater the amount of metal sorbed, the greater the amount available for subsequent acid-driven release. The lowest residual Sc^3+^ concentrations in the desorbing solution were found at ratios 5:1 (11.65 mg/L) and 4:2 (13.20 mg/L), corresponding to compositions that exhibited lower overall sorption capacity for scandium.

As shown in Table 8, the degree of desorption of Sc^3+^ ions varied considerably across the studied hPAA:hP4VP compositions, ranging from 26.42% to 61.03%. The highest desorption efficiencies were recorded at polymer ratios of 5:1 (61.03%), 4:2 (55.85%), and 1:5 (52.00%), indicating that compositions with a clear predominance of either hPAA or hP4VP allow more effective acid-driven release of scandium ions from the polymer matrix. Conversely, the lowest desorption degrees were observed at ratios 2:4 (26.42%) and 3:3 (28.43%)—precisely those compositions that exhibited the highest sorption capacity for Sc^3+^ (Table 6). This inverse relationship between sorption degree and desorption efficiency is consistent with the stronger polymer–ion binding that occurs under conditions of maximum mutual activation of hPAA and hP4VP networks at intermediate ratios, where the enhanced ionization of both polymer chains creates a more stable coordination environment for Sc^3+^ ions that is less readily disrupted by protonation with dilute HNO_3_.

### 2.5. Fourier Transform Infrared (FTIR) Spectroscopy Studies

Fourier-transform infrared (FTIR) spectroscopic analysis was performed on the pristine ion-exchange polymer hydrogels as well as on the polymer samples recovered after the sorption of gallium(III) ions, scandium(III) ions, and their binary mixture. Six samples possessing the best sorption characteristics were selected for investigation: (1) pristine hPAA, (2) pristine hP4VP, (3) hPAA after Ga^3+^ sorption, (4) hP4VP after Ga^3+^ sorption, (5) hPAA after Sc^3+^ sorption, (6) hP4VP after Sc^3+^ sorption, (7) hPAA after sorption from the Ga^3+^/Sc^3+^ binary mixture, and (8) hP4VP after sorption from the Ga^3+^/Sc^3+^ binary mixture. These eight samples (four compositions: 6:0, 0:6, 5:1, 4:2, and 3:3 as applicable, each split into its hPAA and hP4VP fractions) were selected, rather than the full set of seven hPAA:hP4VP ratios, because they correspond to the pristine reference materials (6:0 and 0:6) and to the three compositions exhibiting the highest sorption degrees for Ga^3+^ (5:1 and 3:3) and Sc^3+^ (4:2 and 3:3) identified in Section 2.1, Section 2.2, Section 2.3 and Section 2.4 (Table 2 and Table 6). This targeted selection was adopted to prioritize mechanistic characterization of the compositions of greatest sorption performance and of the two single-component controls, given the practical constraints on FTIR instrument time; it does not imply that the remaining compositions (1:5 and 2:4, beyond those already included) are mechanistically uninformative, and their omission is acknowledged as a limitation of the present spectroscopic dataset. A complete FTIR and TGA/DSC characterization of all seven hPAA:hP4VP compositions would provide a more complete structure–performance correlation and is recommended for future, more exhaustive mechanistic studies.

**Figure 3:**
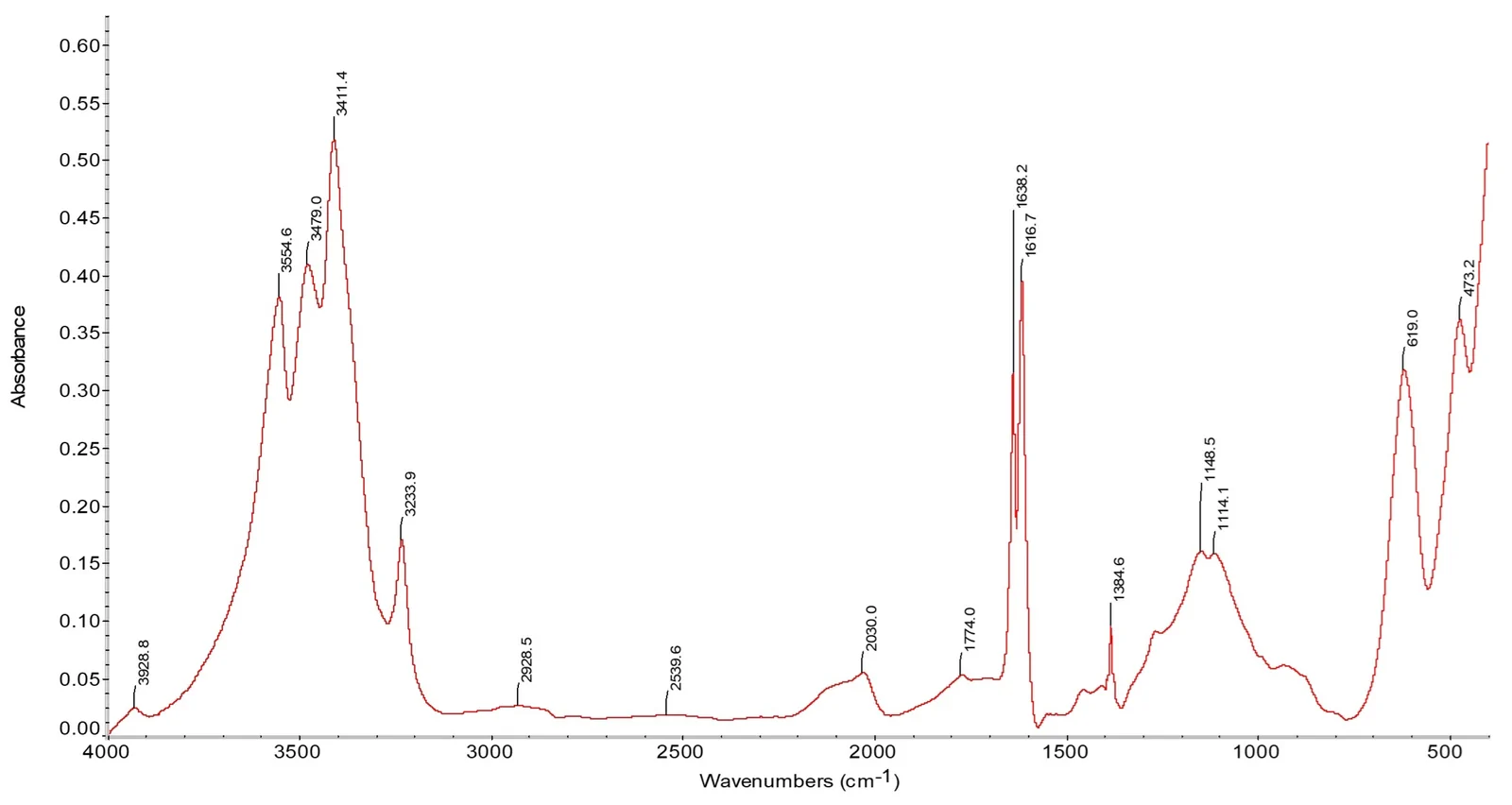
FTIR transmittance spectrum of the pristine polyacrylic acid hydrogel (hPAA) prior to sorption of gallium(III) and scandium(III) ions by the hPAA:hP4VP intergel system.

**Figure 4:**
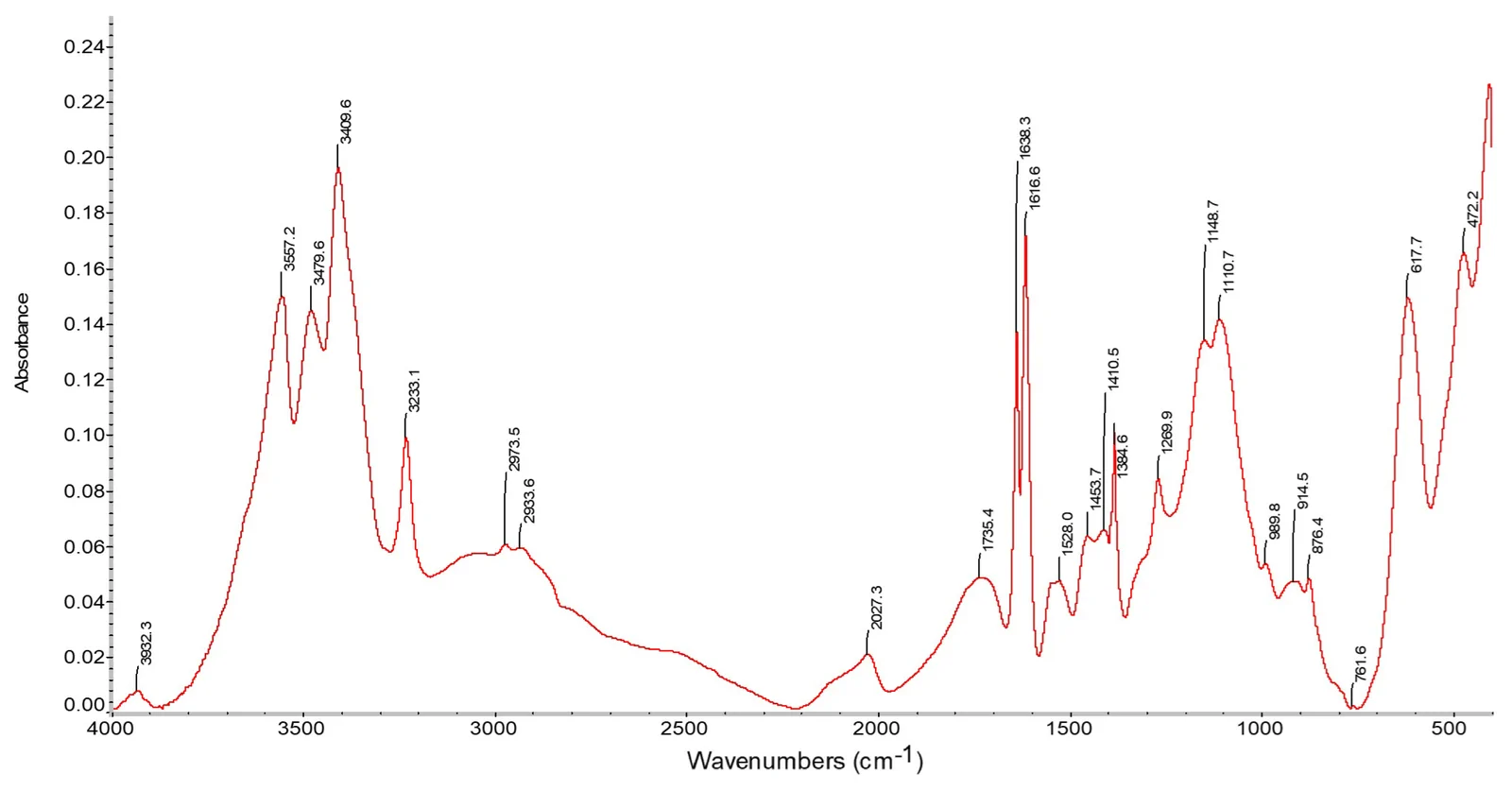
FTIR transmittance spectrum of the polyacrylic acid hydrogel (hPAA) after sorption of gallium(III) and scandium(III) ions by the hPAA:hP4VP intergel system at a polymer mass ratio of 3:3 (50% hPAA: 50% hP4VP).

**Figure 5:**
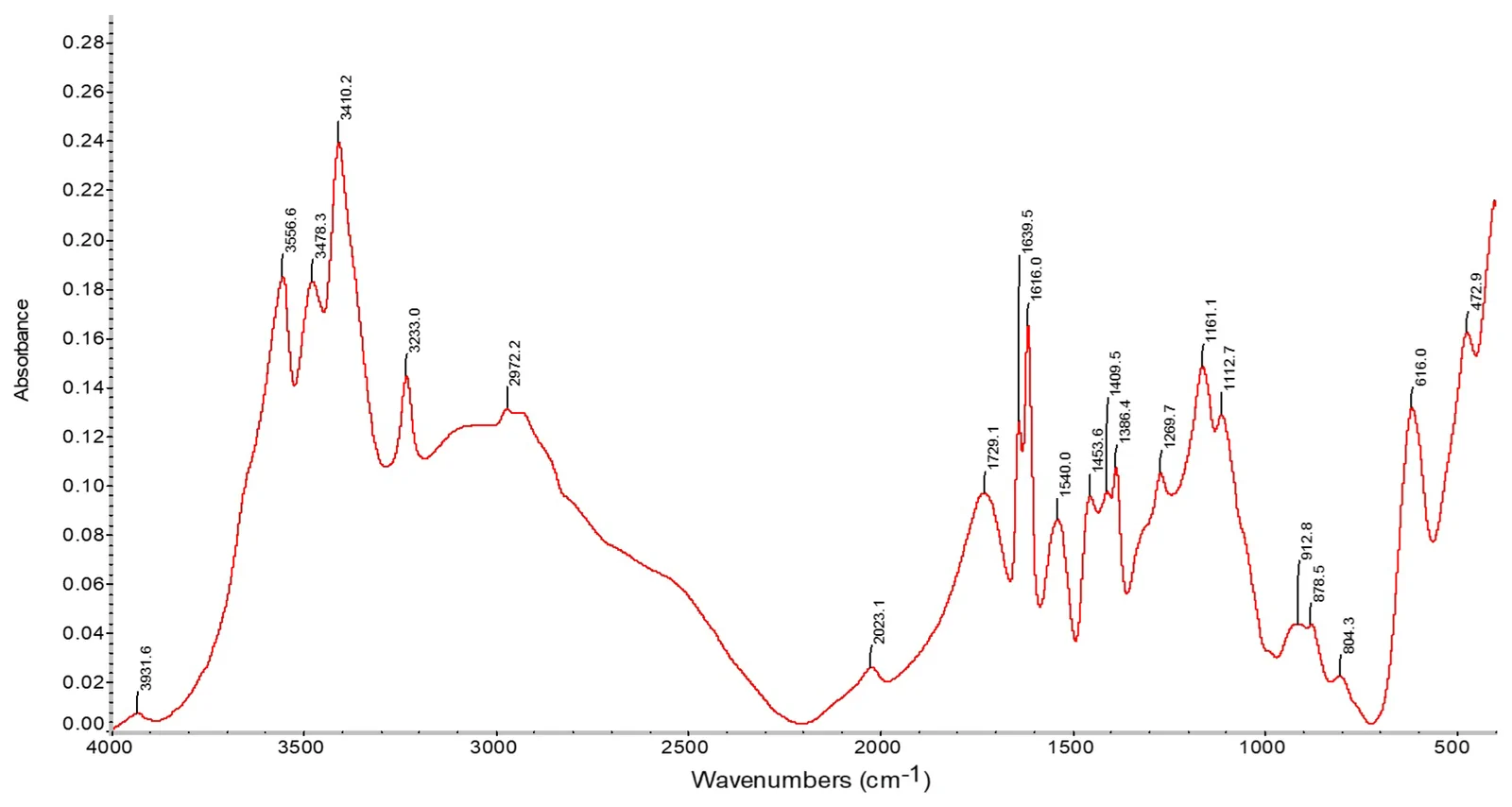
FTIR transmittance spectrum of the polyacrylic acid hydrogel (hPAA) after sorption of scandium(III) ions by the hPAA:hP4VP intergel system at a polymer mass ratio of 4:2 (67% hPAA: 33% hP4VP).

**Figure 6:**
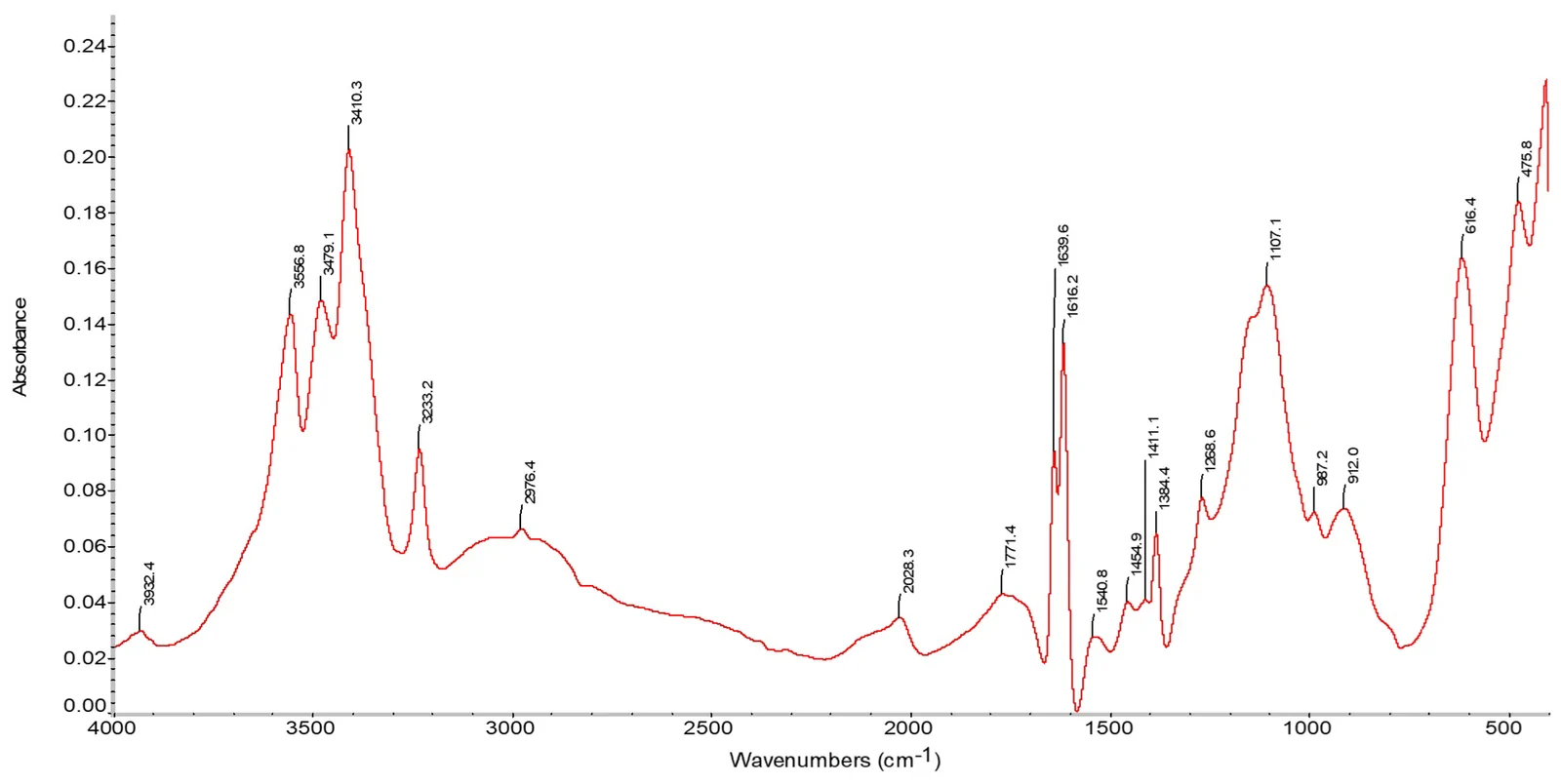
FTIR transmittance spectrum of the polyacrylic acid hydrogel (hPAA) after sorption of gallium(III) ions by the hPAA:hP4VP intergel system at a polymer mass ratio of 5:1 (83% hPAA: 17% hP4VP).

**Figure 7:**
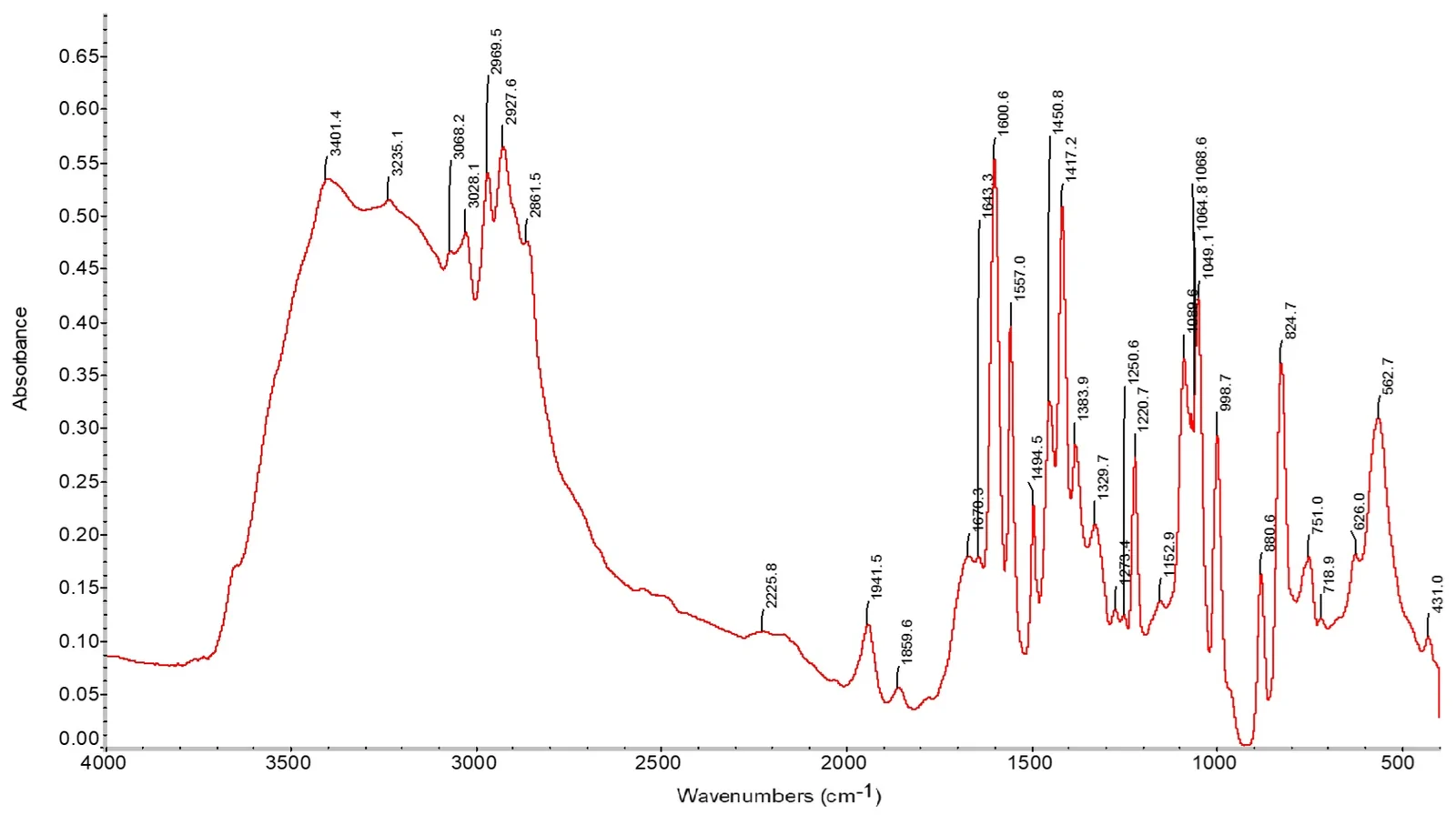
FTIR transmittance spectrum of the pristine poly-4-vinylpyridine hydrogel (hP4VP) prior to sorption of gallium(III) and scandium(III) ions by the hPAA:hP4VP intergel system.

**Figure 8:**
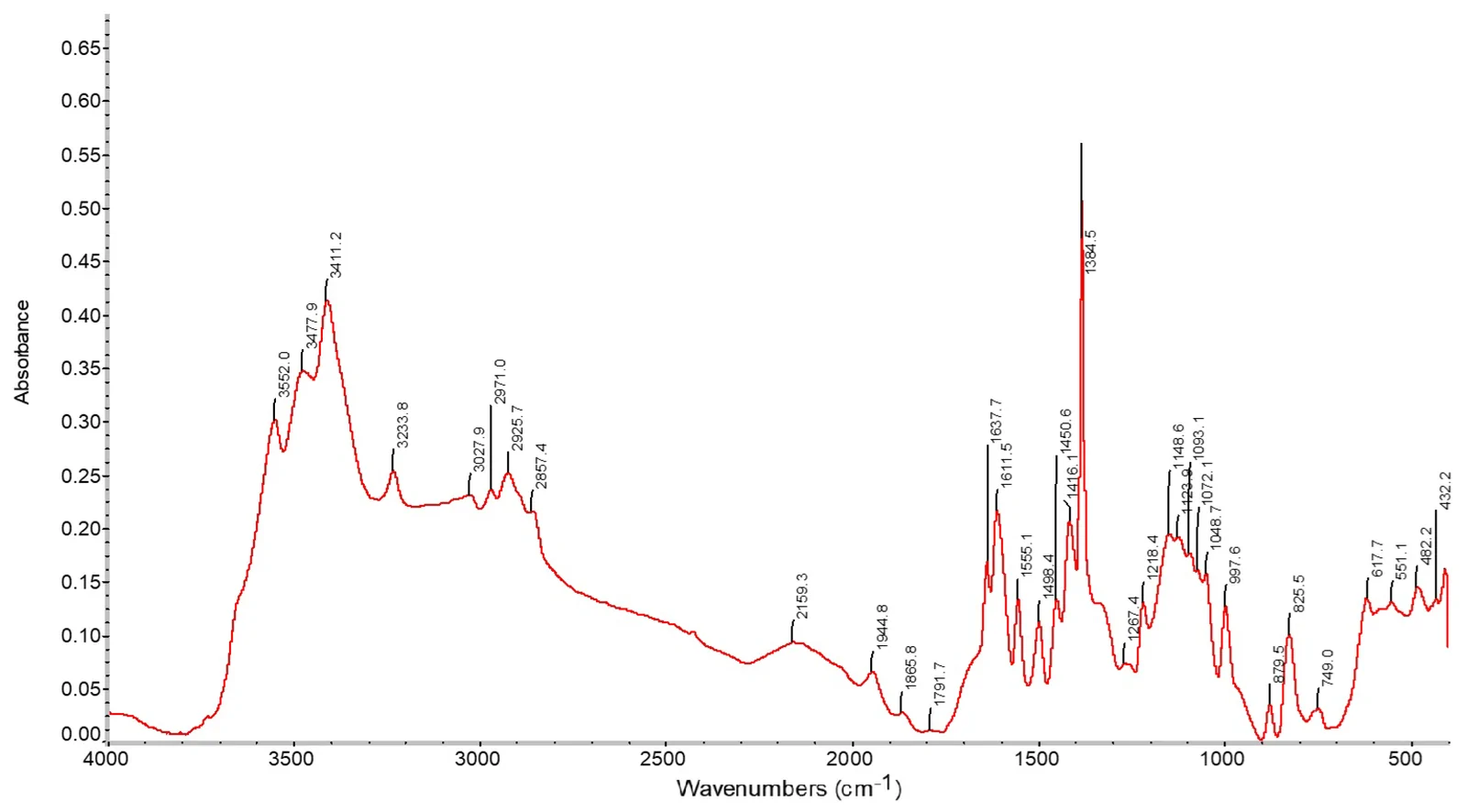
FTIR transmittance spectrum of the poly-4-vinylpyridine hydrogel (hP4VP) after sorption of gallium(III) and scandium(III) ions by the hPAA:hP4VP intergel system at a polymer mass ratio of 3:3 (50% hPAA: 50% hP4VP).

The FTIR transmittance spectra of all indicated samples were recorded in the wavenumber range of 4000–400 cm^−1^ and are presented in Figure 3, Figure 4, Figure 5, Figure 6, Figure 7 and Figure 8. It should be noted that FTIR spectra were collected separately for the hPAA and hP4VP components recovered from each intergel system after remote-contact sorption, rather than for a single spectrum of a physically mixed/co-ground hPAA + hP4VP composite. This approach was adopted deliberately because the intergel design (Section 3.2) relies on spatially separated hydrogels interacting only through the aqueous phase; recording each polymer’s spectrum individually after remote contact allows unambiguous assignment of spectral shifts to the coordination chemistry of that specific polymer (carboxylate vs. pyridine), without spectral overlap or peak superposition that would occur in a single spectrum of a mechanically mixed sample. However, this design does not provide direct spectroscopic evidence of the interpolymer (hPAA···hP4VP) interface itself.

The FTIR analysis was carried out with the aim of identifying spectral shifts and changes in the intensity of characteristic absorption bands that may indicate the formation of coordination bonds between the functional groups of the polymer networks and the sorbed metal ions. For hPAA, the key diagnostic bands include the broad O–H stretching vibration (~3300–2500 cm^−1^), the C=O stretching of carboxylate groups (~1710 cm^−1^ for –COOH and ~1560 cm^−1^ for –COO^−^), and the C–O stretching (~1240 cm^−1^). For hP4VP, the principal diagnostic bands are associated with the pyridine ring stretching vibrations (~1600 and ~1415 cm^−1^) and C–H out-of-plane deformation (~830 cm^−1^), with shifts in the C=N stretching band (~1600 cm^−1^) being particularly informative as evidence of metal coordination through the pyridine nitrogen atom. Because sample thickness, polymer loading, and residual water content were not independently controlled across pristine and metal-loaded pellets, absolute transmittance intensities are not used quantitatively in this study; all intensity comparisons described below (e.g., ‘reduced,’ ‘intensified’) are qualitative and are corroborated primarily by peak-position shifts rather than by intensity magnitude alone.

Figure 3 presents the FTIR transmittance spectrum of the pristine hPAA hydrogel recorded over the range 4000–400 cm^−1^. The spectrum displays several characteristic absorption bands consistent with the structure of crosslinked polyacrylic acid. The broad, intense absorption band in the region of 3500–2500 cm^−1^ is attributed to O–H stretching vibrations of hydrogen-bonded carboxylic groups (–COOH), which are strongly broadened due to intermolecular and intramolecular hydrogen bonding between hydroxyl groups within the polymer network. The prominent band observed at approximately 1600 cm^−1^ corresponds to the asymmetric C=O stretching vibration of the carboxylate anion (–COO^−^), indicating partial deprotonation of acrylic acid units under the measurement conditions. The region 650–400 cm^−1^ represents the fingerprint region, in which the complex pattern of overlapping deformation and skeletal vibration bands is characteristic of the crosslinked polyacrylate backbone.

Figure 4 presents the FTIR spectrum of hPAA recovered after the sorption of Ga^3+^ and Sc^3+^ ions from the binary mixed solution. Comparison with the pristine hPAA spectrum (Figure 4) reveals several spectral changes indicative of metal–polymer interactions. The broad absorption envelope in the region 3600–3200 cm^−1^, with resolved peaks at 3602.3, 3567.2, 3409.6, and 3400.6 cm^−1^, is attributed to O–H stretching vibrations of hydrogen-bonded carboxylic (–COOH) and hydroxyl groups; the persistence of this region after sorption confirms the structural integrity of the polymer network. The band at 2933.6 cm^−1^ corresponds to asymmetric C–H stretching of the –CH– and –CH_2_– groups of the polyacrylate backbone.

A notable feature of the post-sorption spectrum is the band at 1735.4 cm^−1^, assignable to C=O stretching of non-ionized carboxylic groups (–COOH), and the intense overlapping bands at 1638.3 and 1616.6 cm^−1^, corresponding to asymmetric C=O stretching of the carboxylate anion (–COO^−^). The relative increase in intensity in the 1350–1100 cm^−1^ region—with peaks at 1148.7 and 1110.7 cm^−1^—compared to the pristine spectrum reflects enhanced C–O stretching vibrations of the carboxylate group, consistent with the coordination of Ga^3+^ and Sc^3+^ ions to –COO^−^ functional groups and a redistribution of electron density within the carboxylate moiety upon metal binding. The bands at 1463.7, 1410.5, and 1384.6 cm^−1^ in the fingerprint region are attributed to –CH– and –CH_2_– deformation vibrations of the polymer backbone, while the absorption features below 900 cm^−1^ (879.4, 761.6, 617.7, 472.2 cm^−1^) represent skeletal deformation modes characteristic of the crosslinked polyacrylate network. No significant shifts are observed in the O–H stretching region (3600–3200 cm^−1^) or the low-frequency fingerprint region (650–400 cm^−1^) relative to the pristine spectrum, indicating that the overall polymer structure is preserved after the sorption process.

It should be emphasized that the spectral changes described above—including shifts in the carboxylate asymmetric stretching frequency, redistribution of intensity in the O–H stretching envelope, and changes in the 1350–1100 cm^−1^ C–O stretching region—are consistent with, but not uniquely diagnostic of, direct metal–carboxylate coordination. Several alternative or contributing factors could produce similar spectral features: (i) changes in hydrogel swelling degree upon metal loading, which alter the local dielectric environment and hydrogen-bond geometry around carboxylate and hydroxyl groups; (ii) reorganization of the intramolecular and intermolecular hydrogen-bonding network of hPAA independent of metal coordination, driven simply by ionic-strength or pH changes in the surrounding solution; and (iii) differences in residual bound-water content between pristine and metal-loaded samples after the washing/drying steps preceding FTIR measurement, since water itself contributes strongly to the O–H stretching envelope (3600–3200 cm^−1^) and can modulate carbonyl band positions through hydrogen bonding. While the consistent, composition-dependent, and metal-specific character of the band shifts described here (e.g., correlation with the sorption maxima at 3:3 and 4:2 ratios) supports a genuine coordination-driven origin, the present FTIR dataset alone cannot fully exclude these alternative contributions. Complementary techniques—such as swelling-ratio measurements, water-content (moisture) determination by TGA, or comparative FTIR of blank hydrogels equilibrated in metal-free solutions of matched ionic strength—would be required to unambiguously deconvolute coordination-driven shifts from swelling/hydration effects.

Figure 5 presents the FTIR spectrum of hPAA recovered after Sc^3+^ sorption from the binary mixed solution at the 4:2 polymer ratio. Comparison with the pristine hPAA spectrum (Figure 3) reveals several spectral differences indicative of Sc^3+^ coordination with the carboxylate functional groups of the polymer. The broad absorption envelope in the region 3556–3233 cm^−1^ (peaks at 3556.6, 3478.3, 3410.2, and 3233.0 cm^−1^) is attributed to O–H stretching vibrations of hydrogen-bonded carboxylic groups; a slight redistribution of intensity within this envelope compared to the pristine spectrum reflects changes in hydrogen bonding upon Sc^3+^ uptake. The band at 2972.2 cm^−1^ corresponds to C–H stretching of the –CH_2_– groups of the polyacrylate backbone.

Notable differences from the pristine spectrum are observed in the 1800–1100 cm^−1^ region. The band at 1729.1 cm^−1^ is assigned to C=O stretching of non-ionized carboxylic groups (–COOH), and its modified intensity relative to Figure 4 suggests partial deprotonation and involvement of –COOH groups in Sc^3+^ coordination. The overlapping bands at 1639.5 and 1616.0 cm^−1^ correspond to asymmetric C=O stretching of the carboxylate anion (–COO^−^), while the band at 1590.0 cm^−1^ may indicate a shift in the asymmetric carboxylate stretching mode caused by direct metal–oxygen coordination. The region 1500–1100 cm^−1^, with bands at 1483.6, 1380.4, 1209.7, 1161.1, and 1112.7 cm^−1^, encompasses C–O stretching vibrations of the carboxylate group as well as C–H deformation modes of the –CH– and –CH_2_– backbone groups; the altered relative intensities in this region compared to the pristine spectrum are consistent with electron density redistribution upon Sc^3+^ binding to –COO^−^. The fingerprint region below 900 cm^−1^ (899.5, 892.8, 864.3, 616.0, 472.9 cm^−1^) shows skeletal deformation vibrations of the crosslinked polyacrylate network, with no significant structural degradation apparent.

Figure 6 presents the FTIR spectrum of hPAA recovered after Ga^3+^ sorption from the binary mixed solution at the 5:1 polymer ratio. Several characteristic differences from the pristine hPAA spectrum (Figure 3) are evident. The broad absorption envelope in the region 3558–3233 cm^−1^ (peaks at 3558.8, 3479.1, 3410.3, and 3233.2 cm^−1^) corresponds to O–H stretching vibrations of hydrogen-bonded carboxylic groups (–COOH), and its overall profile remains largely preserved relative to the pristine spectrum, confirming the structural integrity of the polymer backbone after Ga^3+^ sorption. The band at 2979.4 cm^−1^ is assigned to asymmetric C–H stretching of the –CH_2_– groups in the polyacrylate chain.

In Figure 6 the most pronounced spectral changes relative to the pristine hPAA are observed in the 1800–900 cm^−1^ region. The band at 1771.4 cm^−1^ is attributed to C=O stretching of non-ionized carboxylic acid groups (–COOH), shifted slightly to higher wavenumbers compared to the typical ~1710–1730 cm^−1^ position, which may reflect changes in the hydrogen-bonding environment of –COOH upon Ga^3+^ coordination. The bands at 1639.6 and 1618.2 cm^−1^ correspond to asymmetric C=O stretching of carboxylate anions (–COO^−^), and the band at 1560.9 cm^−1^ indicates an additional component of the asymmetric –COO^−^ stretching vibration shifted by metal–oxygen bond formation. The region 1500–900 cm^−1^, with peaks at 1454.9, 1441.1, 1448.8, 1394.4, and 1107.1 cm^−1^, encompasses symmetric C=O stretching of –COO^−^ (~1410 cm^−1^), C–O stretching of the carboxylate group (~1250–1100 cm^−1^), and –CH_2_– deformation modes of the polymer backbone. The intensification of bands in this region compared to the pristine spectrum is consistent with enhanced C–O stretching activity associated with Ga^3+^ coordination to carboxylate groups. The fingerprint region below 900 cm^−1^ (912.0, 877.2, 618.4, 479.0 cm^−1^) shows skeletal deformation vibrations of the crosslinked polyacrylate network with no evidence of structural decomposition.

Figure 7 presents the FTIR transmittance spectrum of pristine hP4VP recorded over the range 4000–400 cm^−1^. The spectrum is rich in absorption bands, reflecting the complex structure of the crosslinked poly-4-vinylpyridine network. The absorption region 3535–3401 cm^−1^ (peaks at 3535.1 and 3401.4 cm^−1^) is attributed to O–H stretching vibrations of residual moisture or hydroxyl groups associated with the hydrogel water network, rather than N–H groups, as hP4VP in its neutral (non-protonated) form does not carry N–H bonds. The cluster of bands at 3068.2, 3021.8 cm^−1^ corresponds to aromatic C–H stretching vibrations of the pyridine ring, while the bands at 2969.5, 2861.5, and 2827.6 cm^−1^ are assigned to aliphatic C–H stretching of the –CH– and –CH_2_– groups of the polymer backbone.

The broad continuum absorption observed approximately in the region 3400–2200 cm^−1^, most prominent around 2225.8 cm^−1^, is characteristic of protonated pyridinium species (≡N^+^–H) arising from hydrogen bonding between pyridine nitrogen atoms and residual acidic protons or water molecules in the hydrogel, rather than representing a distinct functional group. The weak combination bands at 1941.5 and 1859.6 cm^−1^ are overtone and combination bands of pyridine ring vibrations, typical of monosubstituted and para-substituted pyridine systems. The highly diagnostic region 1800–1200 cm^−1^ contains multiple characteristic pyridine ring stretching vibrations: the band at 1800.6 cm^−1^ is a pyridine ring overtone, while the bands at 1649.3 and 1557.0 cm^−1^ correspond to pyridine ring C=C and C=N stretching vibrations of free, uncoordinated pyridine rings; the band at ~1595 cm^−1^ in P4VP is well-established as the signature of uncoordinated pyridine rings, while coordination to metal ions causes a shift to ~1620 cm^−1^. The bands at 1484.5 and 1383.9 cm^−1^ are assigned to pyridine ring deformation vibrations, and the band at 1329.7 cm^−1^ is assigned to C–N stretching of the pyridine ring. The region 1220–1050 cm^−1^, with peaks at 1220.7, 1172.1, 1152.9, 1068.6, and 1049.1 cm^−1^, contains in-plane C–H deformation vibrations of the pyridine ring; the bands near 1170–1100 cm^−1^ are particularly characteristic of 4-substituted pyridine systems and serve as diagnostic markers for the pyridine moiety. The fingerprint region 900–400 cm^−1^ (824.7, 800.6, 751.0, 719.9, 626.0, 582.7, 411.0 cm^−1^) contains out-of-plane C–H bending and ring deformation vibrations of the pyridine ring, with the band at ~820 cm^−1^ being a classical out-of-plane C–H deformation band diagnostic for para-substituted pyridine (4-vinylpyridine).

Figure 8 presents the FTIR spectrum of hP4VP recovered after simultaneous sorption of Ga^3+^ and Sc^3+^ ions from the binary mixed solution at the 3:3 polymer ratio. Comparison with the pristine hP4VP spectrum (Figure 7) reveals several meaningful spectral changes indicative of metal–pyridine coordination.

A notable decrease in absorption intensity is observed in the broad region 3500–2800 cm^−1^ (peaks at 3477.9, 3411.2, 3233.8, 2971.0, 2925.7, and 2857.4 cm^−1^) relative to the pristine spectrum. This region encompasses O–H stretching of hydrogel-bound water and aromatic/aliphatic C–H stretching vibrations of the pyridine ring and polymer backbone; the reduced intensity may reflect partial dehydration or rearrangement of the hydrogen-bonding network within the polymer upon metal ion uptake. The broad continuum around 2159.3 cm^−1^, representing the hydrogen-bonded pyridinium species (≡N···H), is markedly diminished compared to the pristine spectrum, suggesting that pyridine nitrogen atoms are increasingly engaged in direct coordination with Ga^3+^ and Sc^3+^ rather than in hydrogen bonding with water.

The most diagnostically significant changes occur in the 1650–1400 cm^−1^ region, where the intensities of bands at 1637.7, 1611.5, 1555.1, 1494.4, and 1450.6 cm^−1^ are substantially reduced (approximately by a factor of two) relative to Figure 7. These bands correspond to pyridine ring C=C and C=N stretching vibrations and ring deformation modes; their intensity reduction and the upward shift in the C=N stretching band from ~1557 cm^−1^ toward ~1611–1637 cm^−1^ are hallmark spectral signatures of pyridine nitrogen coordination to metal ions (Ga^3+^ and Sc^3+^), consistent with published data for metal-loaded P4VP systems. The most intense band in the post-sorption spectrum appears at 1384.5 cm^−1^, assigned to –CH_2_– scissoring deformation of the polymer backbone; its relative prominence after sorption reflects the overall decrease in pyridine ring band intensities rather than an absolute increase in the 1384.5 cm^−1^ band itself. The region 1170–1100 cm^−1^ (peaks at 1146.6, 1072.1, 1053.1, 1045.7 cm^−1^)—diagnostic for 4-substituted pyridine in-plane C–H deformation—also shows a ca. two-fold reduction in intensity, further confirming the involvement of the pyridine ring system in metal coordination. The fingerprint region 900–430 cm^−1^ (825.5, 829.5, 749.0, 617.7, 482.2, 432.2 cm^−1^) similarly displays reduced intensities, consistent with changes in ring deformation modes upon metal binding. As discussed in Section 2.5 for hPAA, the upward shift in the pyridine C=N/ring-stretching band (~1557→~1611–1637 cm^−1^) is consistent with metal coordination but cannot be fully distinguished from a shift caused by variable pyridinium (≡N^+^–H) protonation, since both effects perturb the same ring π-system in the same direction; the composition-dependent correlation of this shift with sorption maxima (Section 2.8) is therefore the stronger evidential basis for coordination than the shift itself.

### 2.6. Thermogravimetric Analysis of Polymer Samples

**Figure 9:**
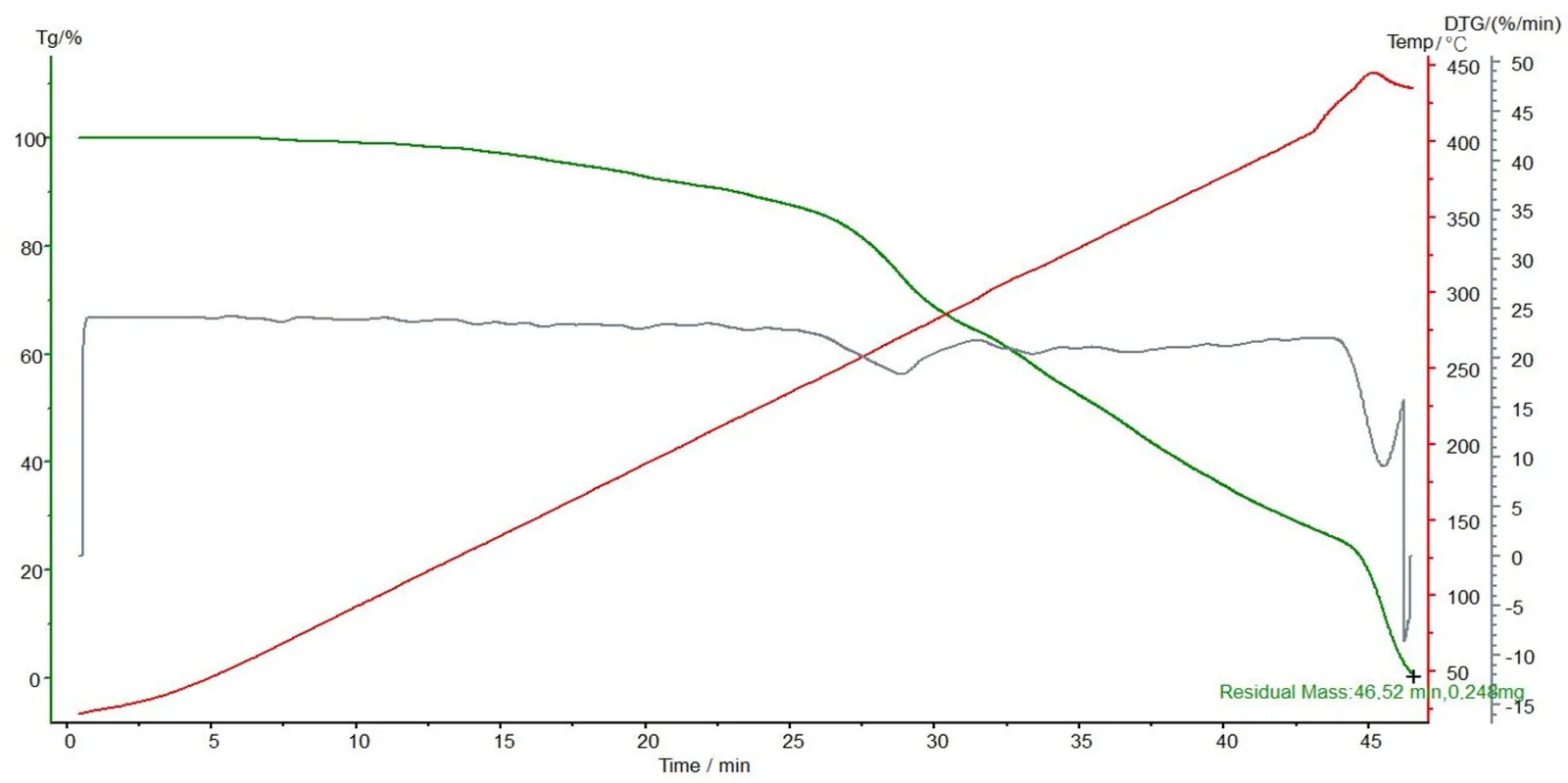
DTG curve of the pristine hPAA ion-exchange polymer hydrogel prior to sorption of gallium(III) and scandium(III) ions: mass loss rate as a function of temperature and residence time in the crucible. The sign “+” indicates the exact points where the measurements were taken.

**Figure 10:**
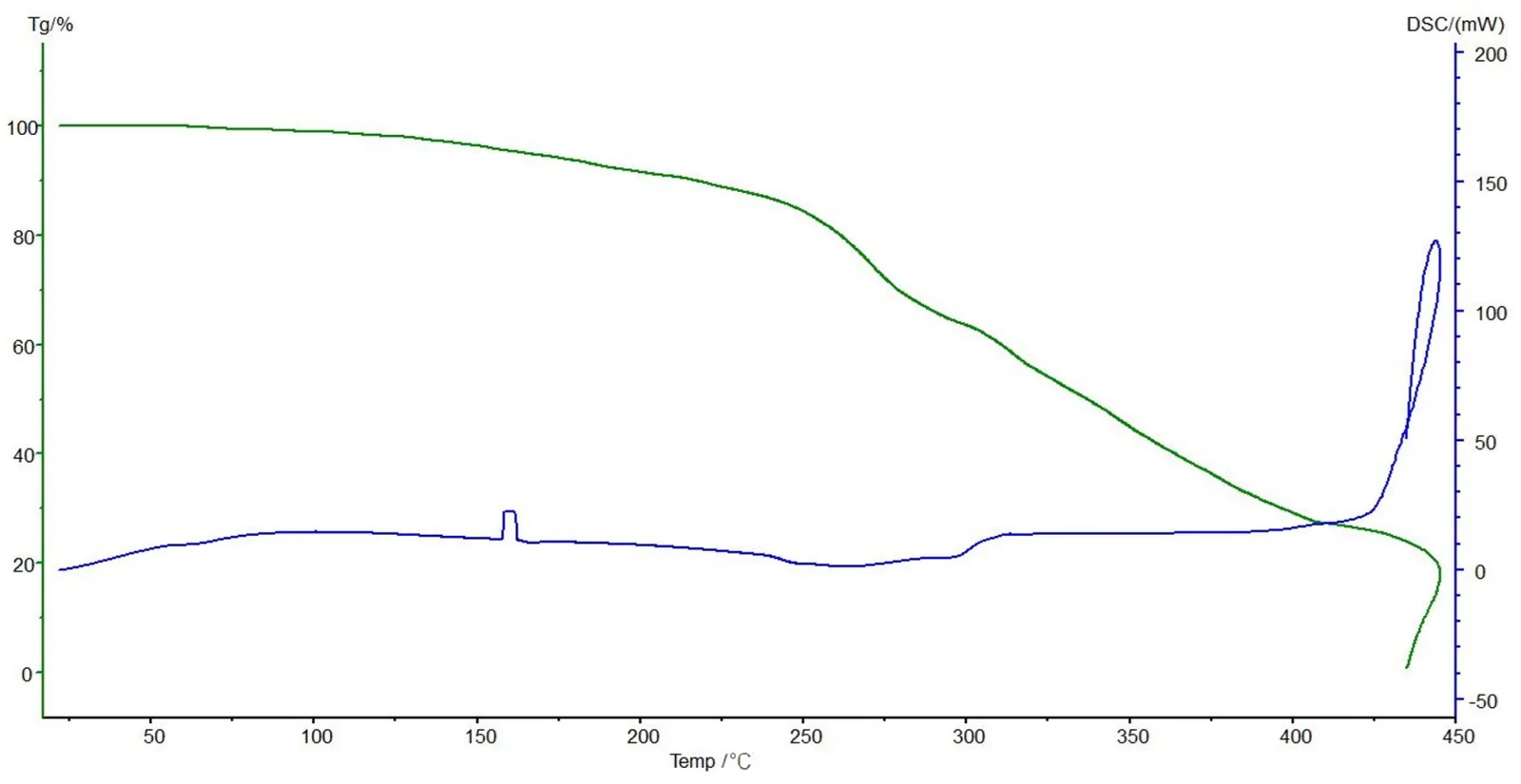
DSC curve of the pristine polyacrylic acid hydrogel (hPAA) prior to sorption of gallium(III) and scandium(III) ions: heat flow as a function of temperature and residence time in the crucible.

**Figure 11:**
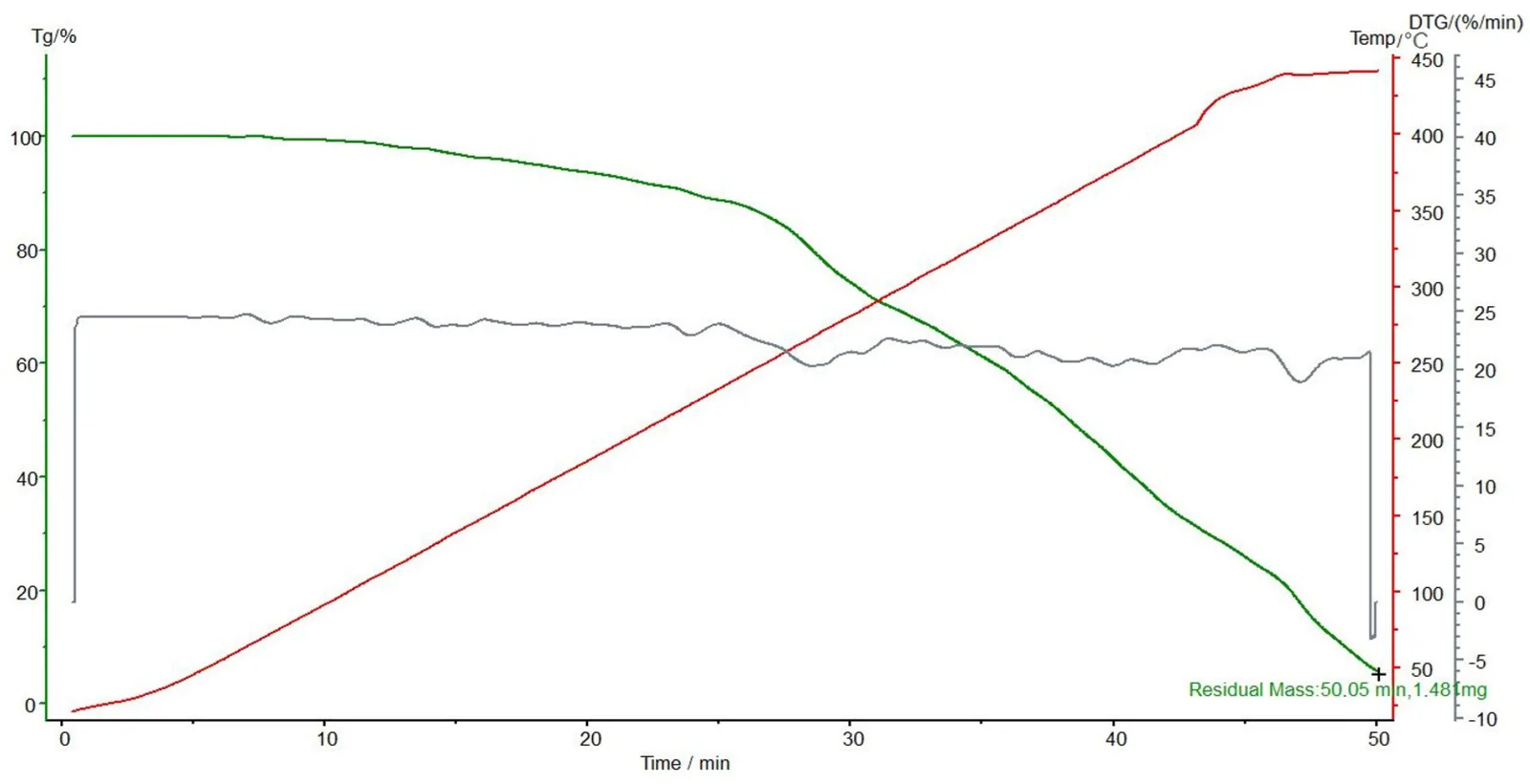
DTG curve of the hPAA hydrogel after sorption of gallium(III) and scandium(III) ions from the binary mixed solution by the hPAA:hP4VP intergel system (ratio 3:3; 50% hPAA: 50% hP4VP): mass loss rate as a function of temperature and residence time in the crucible.

**Figure 12:**
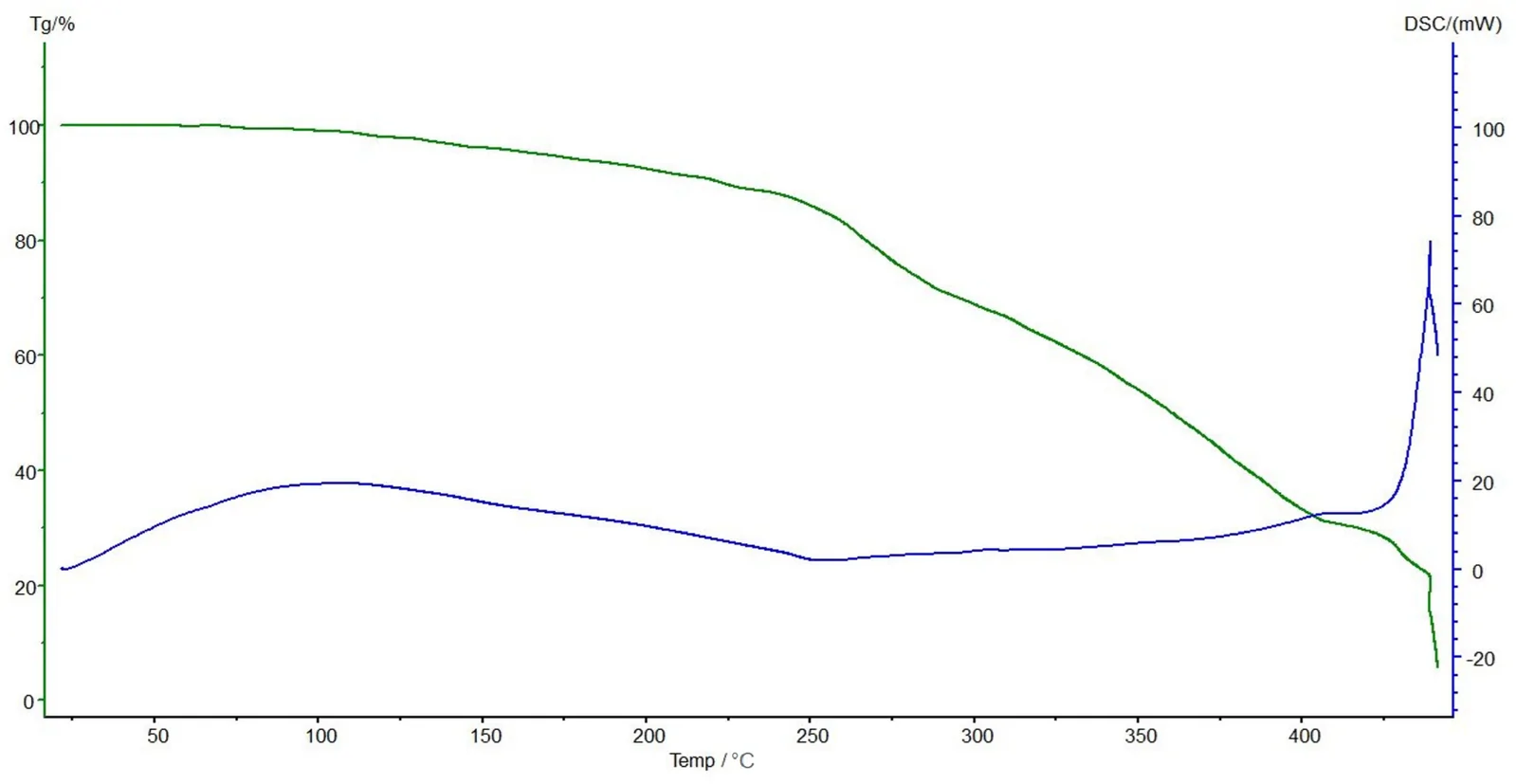
DSC curve of the hPAA hydrogel after sorption of gallium(III) and scandium(III) ions from the binary mixed solution by the hPAA:hP4VP intergel system (ratio 3:3; 50% hPAA: 50% hP4VP): heat flow as a function of temperature and residence time in the crucible.

**Figure 13:**
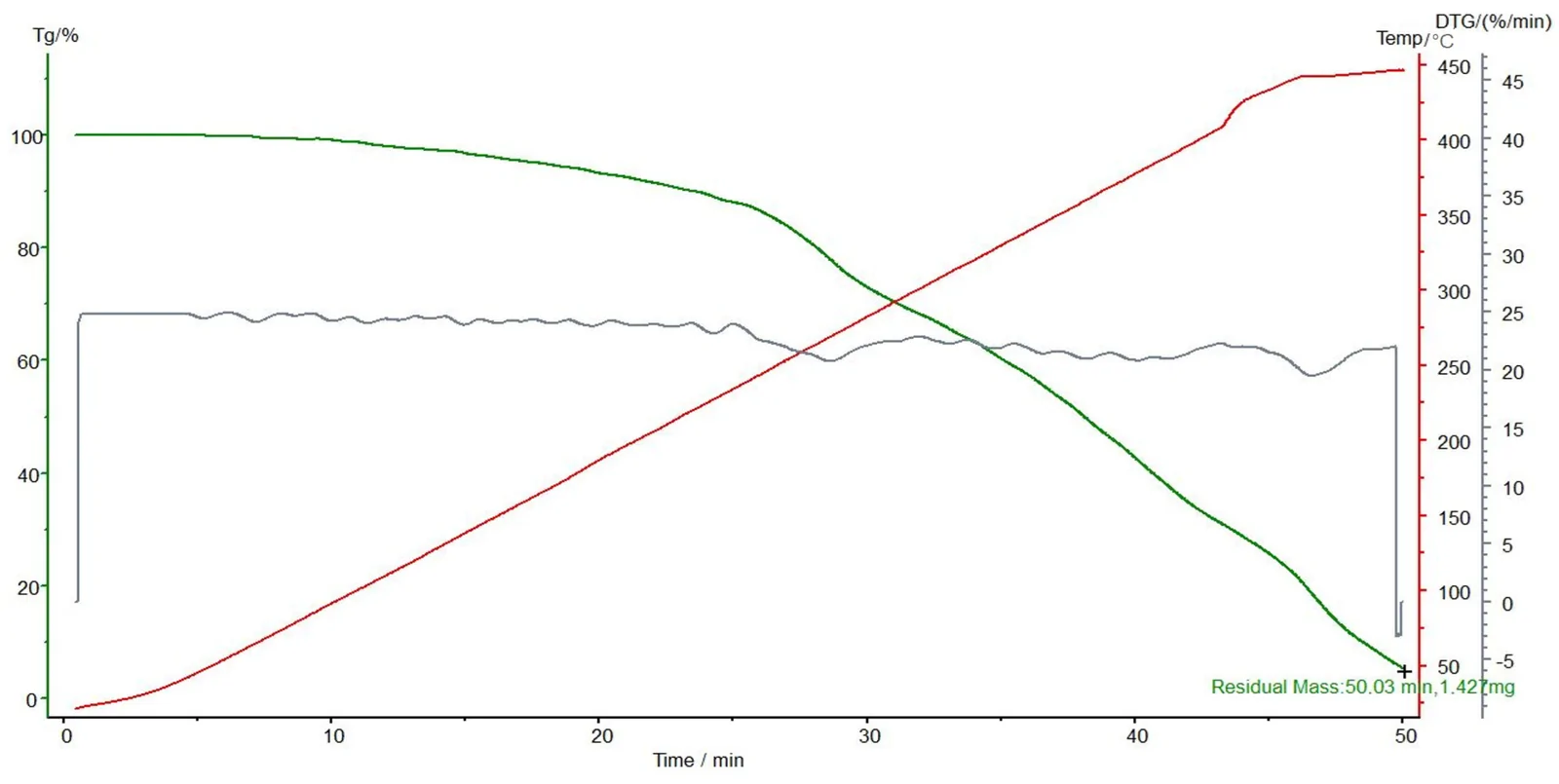
DTG curve of the hPAA hydrogel after sorption of scandium(III) ions by the hPAA:hP4VP intergel system (ratio 2:4; 33%hPAA: 67% hP4VP): mass loss rate as a function of temperature and residence time in the crucible.

**Figure 14:**
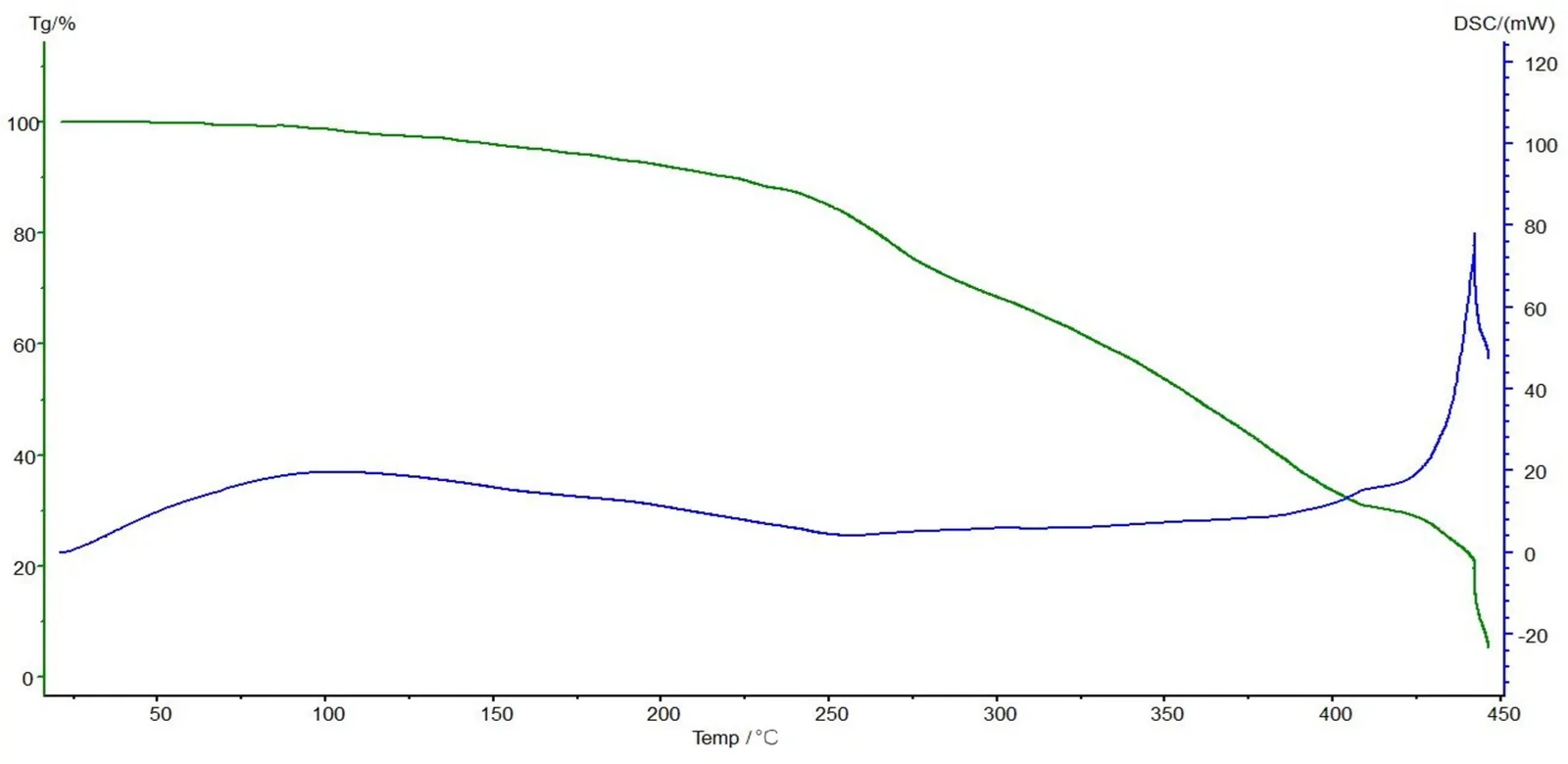
DSC curve of the hPAA hydrogel after sorption of scandium(III) ions by the hPAA:hP4VP intergel system (ratio 2:4; 33%hPAA: 67% hP4VP): heat flow as a function of temperature and residence time in the crucible.

**Figure 15:**
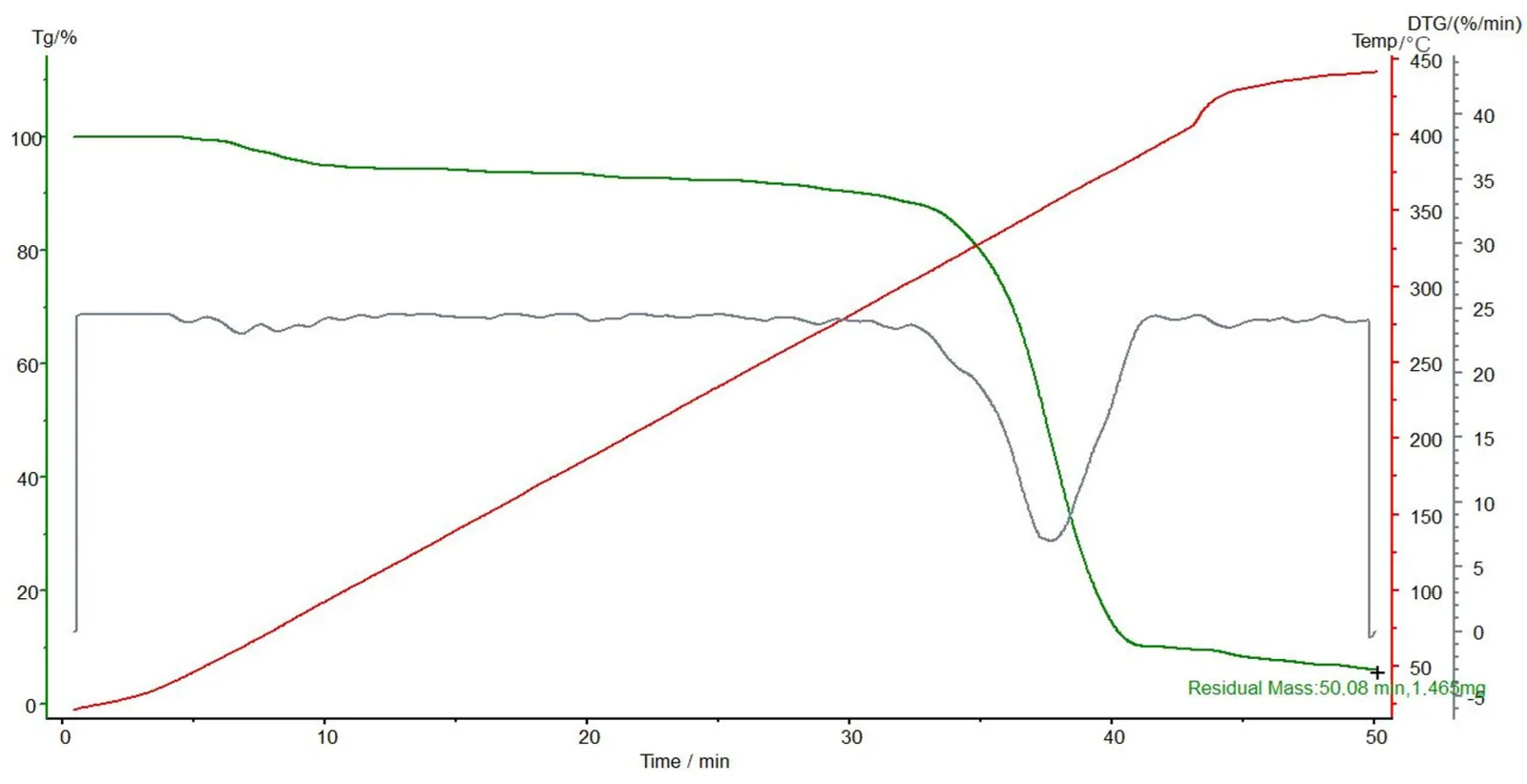
DTG curve of the pristine hP4VP ion-exchange polymer hydrogel prior to sorption of gallium(III) and scandium(III) ions by the hPAA:hP4VP intergel system: mass loss rate as a function of temperature and residence time in the crucible.

**Figure 16:**
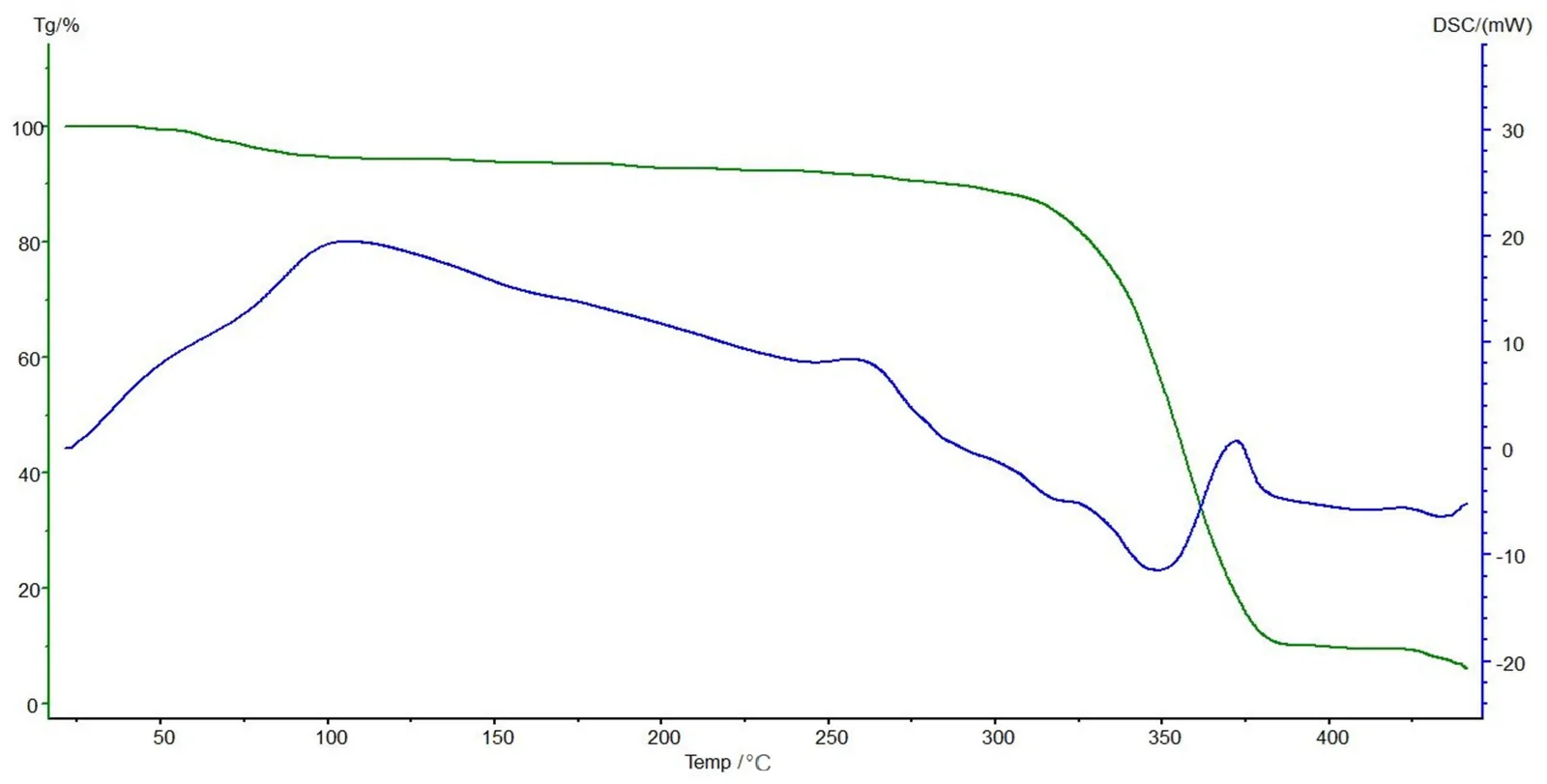
DSC curve of the pristine poly-4-vinylpyridine hydrogel (hP4VP) prior to sorption of gallium(III) and scandium(III) ions by the hPAA:hP4VP intergel system: heat flow as a function of temperature and residence time in the crucible.

**Figure 17:**
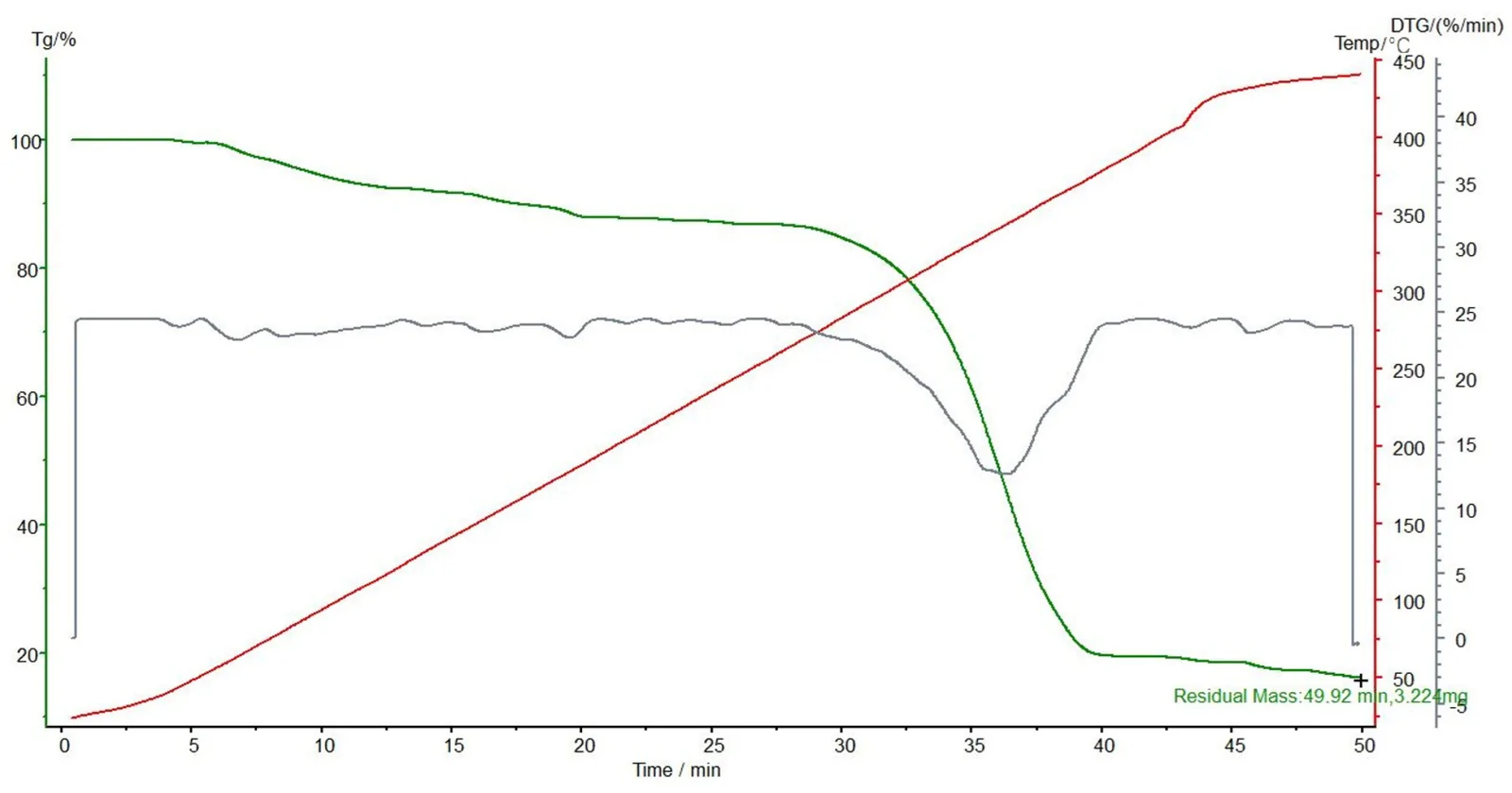
DTG curve of the hP4VP hydrogel after sorption of gallium(III) and scandium(III) ions from the binary mixed solution by the hPAA:hP4VP intergel system (ratio 3:3; 50% hPAA: 50% hP4VP): mass loss rate as a function of temperature and residence time in the crucible.

**Figure 18:**
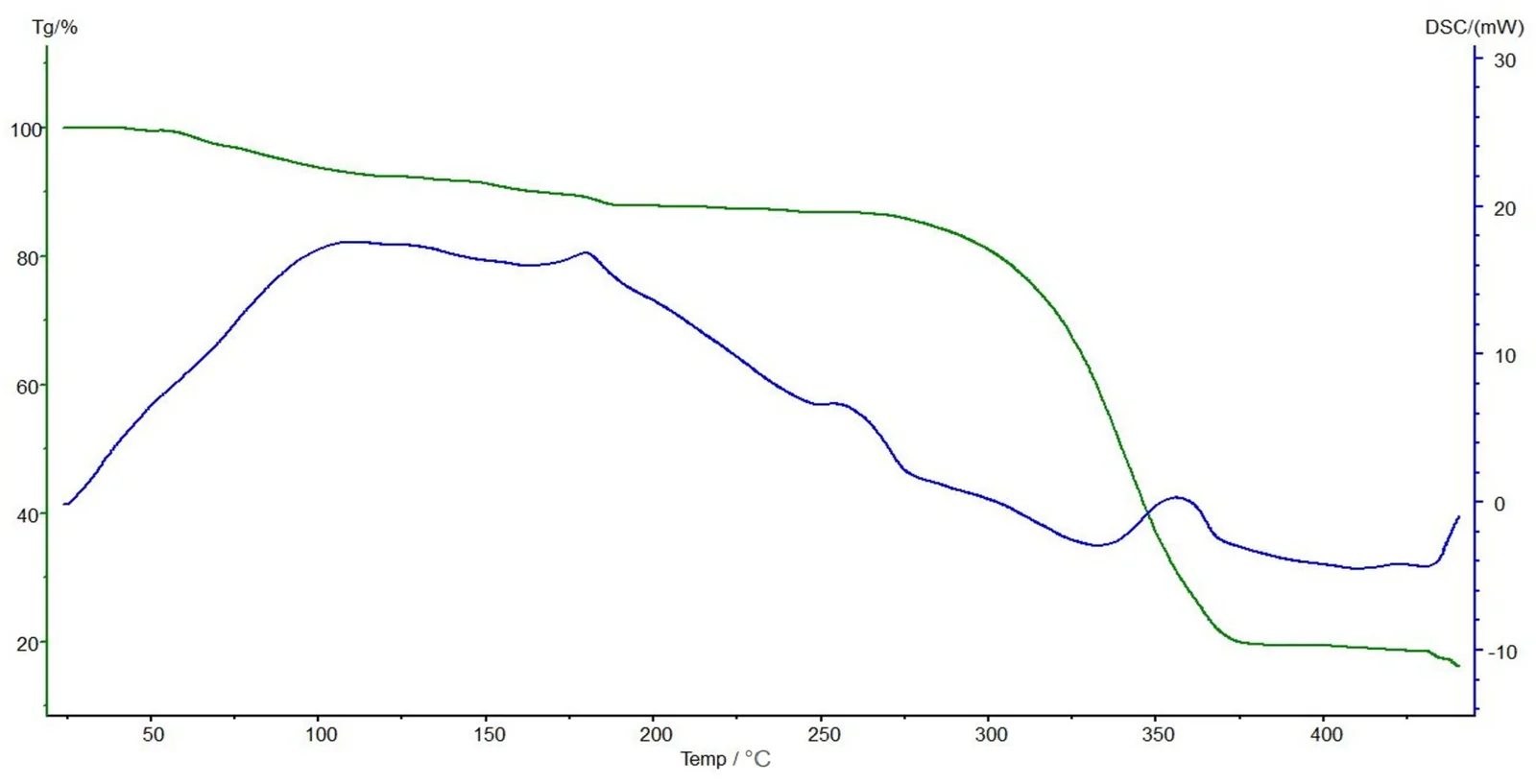
DSC curve of the hP4VP hydrogel after sorption of gallium(III) and scandium(III) ions from the binary mixed solution by the hPAA:hP4VP intergel system (ratio 3:3; 50% hPAA: 50% hP4VP): heat flow as a function of temperature and residence time in the crucible.

Thermogravimetric analysis (TGA) combined with differential scanning calorimetry (DSC) was performed on pristine ion-exchange polymer hydrogels and on polymers recovered after the sorption of gallium(III) ions, scandium(III) ions, and their binary mixture. All measurements were carried out under a controlled heating rate of 10 °C/min up to a maximum temperature of 450 °C. The following samples were selected for analysis based on their best sorption characteristics: (1) pristine hPAA, (2) pristine hP4VP, (3) hPAA and hP4VP after sorption from the Ga^3+^/Sc^3+^ binary mixed solution at the 3:3 ratio (50% hPAA: 50% hP4VP), (4) hPAA after Sc^3+^ sorption at the 4:2 ratio (67% hPAA: 33% hP4VP), and (5) hPAA after Ga^3+^ sorption at the 5:1 ratio (83% hPAA: 17% hP4VP). As in the FTIR analysis (Section 2.5), these five samples were selected from among the seven possible hPAA:hP4VP compositions because they represent the pristine reference materials and the compositions exhibiting the highest sorption degrees for each metal individually and for the binary mixture (Table 2 and Table 6), thereby prioritizing thermal characterization of the most sorption-relevant systems within the available instrument time; extension of the TGA/DSC dataset to the remaining compositions (5:1 for Sc-only, 1:5, and 2:4 for Ga-only, etc.) is recommended for future work to establish a complete thermal structure–composition relationship. The TGA, DTG (mass loss rate), and DSC curves for all samples are presented in Figure 9, Figure 10, Figure 11, Figure 12, Figure 13, Figure 14, Figure 15, Figure 16, Figure 17 and Figure 18.

#### 2.6.1. TG/DTG/DSC Analyses of hPAA (6:0)

Figure 9 presents the combined TGA and DTG curves of pristine hPAA. The DTG curve—reflecting the rate of mass loss as a function of temperature and residence time—remains nearly constant throughout the majority of the heating program, indicating a steady, gradual mass reduction without discrete sharp decomposition steps preceding the main event. A sharp decrease in the mass loss rate is observed only at the end of the process, consistent with near-total polymer decomposition, after which no further significant mass is available for degradation.

Figure 10 shows the TGA/DSC curve of pristine hPAA. The DSC signal remains essentially constant until the final stage of the experiment, after which a sharp increase in heat flow is observed at ~450 °C, corresponding to the terminal decomposition of the polymer complex. This behavior is consistent with the primarily single-stage thermal degradation observed in the TGA profile.

#### 2.6.2. TG/DTG/DSC Analyses of hPAA After Sorption from Ga^3+^/Sc^3+^ Binary Mixed Solution (3:3 Ratio)

Figure 11 presents the combined TGA/DTG curves for this sample. As with the pristine polymer, the DTG curve shows a nearly constant mass loss rate throughout the process, with a sharp decrease at the end corresponding to near-complete polymer degradation. No additional decomposition steps or rate anomalies are introduced by metal loading, consistent with the minor structural changes indicated by FTIR analysis.

Figure 12 shows the TGA/DSC profile of hPAA after Ga^3+^/Sc^3+^ sorption at the 3:3 ratio. In contrast to the pristine polymer, a slight difference is observed at the beginning of the heating process: the heat flow initially increases before gradually decreasing toward the end of the experiment. This early-stage deviation may reflect the additional thermal energy required to disrupt metal–carboxylate coordination bonds between Ga^3+^/Sc^3+^ and the –COO^−^ groups of hPAA before backbone decomposition proceeds. The sharp increase in heat capacity at the terminal stage (~450 °C) is consistent with near-complete polymer decomposition, as observed for the pristine sample.

#### 2.6.3. TG/DTG/DSC Analyses of hPAA After Sc^3+^ Sorption (2:4 Ratio)

Figure 13 shows the TGA/DTG curve for this sample. The DTG profile mirrors that of the pristine polymer—a nearly constant mass loss rate throughout, followed by a sharp decline at the end as decomposition reaches completion. No new intermediate decomposition stages are introduced by Sc^3+^ coordination, supporting the conclusion that scandium binding to –COO^−^ groups does not substantially modify the bulk thermal behavior of the hPAA network.

Figure 14 presents the TGA/DSC curve of hPAA after Sc^3+^ sorption. The heat capacity profile is essentially coincident with that of the binary sorption sample: heat capacity increases initially, then gradually decreases through the main decomposition range, with a sharp rise at the terminal stage. The consistency between the DSC profiles of all three metal-loaded hPAA samples (Figure 13) suggests that the perturbation of thermal properties by metal coordination is qualitatively similar regardless of whether Ga^3+^, Sc^3+^, or both are sorbed.

#### 2.6.4. TG/DTG/DSC Analyses of Pristine hP4VP (0:6)

Figure 15 presents the combined TGA/DTG curves of pristine hP4VP. The DTG curve shows a sharp, well-defined decrease in mass loss rate at ~350 °C and 35 min residence time, followed by a partial recovery of the rate (~40 min) before the terminal sharp decrease marking near-complete decomposition. This bimodal DTG feature—absent in hPAA—reflects the two-stage character of P4VP thermal degradation: (i) depolymerization and volatilization of pyridine-containing oligomers, followed by (ii) carbonization and decomposition of the residual crosslinked matrix.

Figure 16 shows the TGA/DSC curve of pristine hP4VP. The heat capacity increases in the early stages of the experiment, then decreases gradually, with minor fluctuations near ~380 °C corresponding to the onset of terminal polymer decomposition. This DSC behavior is distinct from that of hPAA, reflecting the different thermal stability and decomposition chemistry of the pyridine-based polymer network.

#### 2.6.5. TG/DTG/DSC Analyses of hP4VP After Sorption from Ga^3+^/Sc^3+^ Binary Mixed Solution (3:3 Ratio)

Figure 17 presents the TGA/DTG curves of metal-loaded hP4VP. The DTG profile closely mirrors that of pristine hP4VP (Figure 15): a sharp decrease in mass loss rate at ~350 °C and ~35 min, followed by partial rate recovery at ~40 min and a terminal sharp decline. The preservation of the bimodal DTG character after metal loading indicates that the fundamental two-stage decomposition mechanism of hP4VP is retained, although the slightly earlier onset (30 vs. 35 min) may indicate moderate weakening of interchain interactions by Ga^3+^/Sc^3+^ coordination with pyridine nitrogen atoms.

Figure 18 presents the TGA/DSC curve of hP4VP after binary sorption. The heat capacity profile closely parallels that of pristine hP4VP (Figure 17), with an initial increase followed by a gradual decrease and minor fluctuations at ~340 °C—slightly lower than the ~380 °C observed for the pristine sample. This modest shift in the DSC feature to lower temperature upon metal loading is consistent with partial disruption of the pyridine ring π-system and interchain stacking interactions as a result of Ga^3+^ and Sc^3+^ coordination, in agreement with the FTIR evidence for metal–pyridine coordination.

### 2.7. Comparative Summary of TGA Results

The TGA/DTG/DSC results for all hPAA and hP4VP samples—pristine and metal-loaded—are collectively consistent with the following conclusions:

Thermal stability of hPAA is not significantly affected by Ga^3+^ or Sc^3+^ sorption: the decomposition onset temperature (~350 °C) and overall mass loss profile are nearly identical for all hPAA samples regardless of metal loading or polymer ratio. This indicates that carboxylate–metal coordination bonds are of insufficient thermal stability to shift the main polymer degradation temperature under these measurement conditions.

Thermal behavior of hP4VP shows slightly more sensitivity to metal loading: the minor shift in the DTG peak and DSC feature to lower temperatures (~5–10 °C earlier) in the binary-loaded hP4VP sample suggests that Ga^3+^/Sc^3+^ coordination with pyridine nitrogen introduces subtle structural perturbations in the polymer network, consistent with the FTIR evidence for C=N band shifts upon metal binding.

DSC analysis reveals a consistent difference between pristine and metal-loaded hPAA: the initial increase in heat capacity at the beginning of the heating program—observed for all metal-loaded hPAA samples but not for pristine hPAA—may be attributed to the endothermic dissociation of metal–carboxylate coordination complexes prior to the onset of bulk polymer chain decomposition.

Both hPAA and hP4VP retain their bulk structural and thermal integrity up to approximately 320–340 °C. For hP4VP specifically, the DTG profile (Figure 15) identifies the onset of the first sharp, well-defined decomposition event at ~350 °C; therefore, the thermally stable operating window for hP4VP-containing compositions should be understood as extending up to, but not beyond, ~320–340 °C, with decomposition becoming significant immediately above this range. This distinction is important because the earlier statement in the Abstract and Conclusions describing stability “up to ~350 °C” refers to the onset of the hP4VP decomposition event itself rather than to a temperature at which the polymer remains fully stable; the two figures (~320–340 °C bulk stability vs. ~350 °C decomposition onset) are therefore consistent once precisely defined, rather than contradictory.

### 2.8. Preferential Uptake and Compositional Tuning of Ga(III)/Sc(III) Sorption

The hPAA:hP4VP intergel system shows pronounced compositional dependence in its uptake of Ga^3+^ and Sc^3+^, with distinct optimal ratios for each metal (Section 2.1, Section 2.2, Section 2.3 and Section 2.4): Ga^3+^ sorption is maximized at the equimolar 3:3 ratio, while Sc^3+^ sorption is maximized at the hP4VP-rich 2:4 ratio. This divergence is consistent with the Hard–Soft Acid–Base (HSAB) principle: Ga^3+^ (ionic radius 0.62 Å) is a harder acid than Sc^3+^ (ionic radius 0.745 Å) and therefore shows a stronger preference for the hard oxygen donors of carboxylate groups in hPAA-rich compositions, whereas the somewhat softer, larger Sc^3+^ ion tolerates mixed O/N coordination more readily, favoring compositions with greater hP4VP content.

The competitive sorption from the binary Ga^3+^/Sc^3+^ mixed solution reveals a synergistic effect of the intergel system: both metals are sorbed simultaneously, with neither completely dominating the available coordination sites. However, at the metal-specific optimal ratios (3:3 for Ga^3+^, 2:4 for Sc^3+^), the system exhibits preferential enrichment of the corresponding metal, suggesting a basis for sequential or selective separation of Ga^3+^ and Sc^3+^ by tuning the polymer composition.

Desorption selectivity further supports differential binding: the lowest desorption degrees for Sc^3+^ (26.42% and 28.43% at ratios 2:4 and 3:3 respectively) correspond precisely to the compositions of highest Sc^3+^ sorption, confirming stronger metal–polymer binding at these ratios. In contrast, Ga^3+^ desorption at the optimal 3:3 ratio is more facile, indicating a somewhat weaker average binding energy relative to Sc^3+^ at equivalent compositions. This divergence in desorption behavior between Ga^3+^ and Sc^3+^ confirms that the intergel system not only distinguishes between the two metals during sorption but also during regeneration, which has important implications for the design of cyclic sorption–desorption separation processes.

### 2.9. Sorption Mechanism

The combined evidence from sorption experiments, FTIR spectroscopy, and TGA/DSC analysis allows a coherent, evidence-based mechanistic hypothesis to be proposed for the sorption of Ga^3+^ and Sc^3+^ by the hPAA:hP4VP intergel system; direct quantitative confirmation of the proposed mutual activation step (via potentiometric titration, swelling-ratio, and charge-density measurements, as discussed below) remains an important target for future work.

Stage 1. Mutual activation of the intergel system. Upon mixing hPAA and hP4VP hydrogels in aqueous solution, an intermacromolecular proton transfer occurs from the –COOH groups of hPAA to the pyridine nitrogen atoms of hP4VP. This equilibrium deprotonation of hPAA generates –COO^−^ carboxylate anions—the primary active coordination sites for metal cations—while simultaneously protonating hP4VP to form pyridinium species (≡N^+^H), which restructure the electrostatic environment of the hP4VP network. The degree of mutual activation is maximized at intermediate polymer ratios (3:3 to 4:2), consistent with the observation that maximum sorption of both metals occurs in this compositional range.

The mutual activation mechanism proposed above is presently supported by indirect evidence—the composition dependence of sorption degree and Kd (Section 2.10) and the qualitative FTIR band changes (Section 2.5)—rather than by direct, quantitative measurements of the ionization state of hPAA and hP4VP within the intergel system. A rigorous, quantitative test of the mutual activation hypothesis would require: (i) potentiometric titration of hPAA and hP4VP individually versus in the intergel configuration, to determine whether the apparent pKa of hPAA decreases and the apparent pKa of the hP4VP pyridinium equilibrium shifts upon remote contact, as predicted by the proton-transfer mechanism in Equations (1) and (2); (ii) gravimetric or volumetric swelling-ratio measurements of each hydrogel before and during remote contact, since enhanced ionization is expected to increase electrostatic repulsion within each polyelectrolyte network and thereby increase its equilibrium swelling ratio; and (iii) direct or indirect estimation of the surface/bulk charge density of each hydrogel (e.g., via ion-exchange capacity titration or conductometric titration, as noted for the electrical conductivity measurements in Section 3.3) under intergel versus isolated conditions.

Stage 2. Electrostatic attraction and outer-sphere complex formation. The highly charged –COO^−^ groups and the restructured hP4VP network attract Ga^3+^ and Sc^3+^ cations from solution through long-range electrostatic interactions. At this stage, metal ions enter the swollen hydrogel network accompanied by their hydration shells, forming outer-sphere (electrostatic) complexes with the anionic sites of hPAA.

Stage 3. Inner-sphere coordination bond formation. As metal ions diffuse deeper into the polymer network and residence time increases, partial or complete dehydration of the metal coordination sphere occurs, enabling direct coordination bond formation between the metal center and the functional groups of the polymer. For hPAA, the FTIR evidence—shifts in the asymmetric C=O stretching of –COO^−^ from ~1616 cm^−1^ toward higher wavenumbers, intensification of C–O stretching bands at 1148–1110 cm^−1^, and modification of the 1729–1735 cm^−1^ C=O band—confirms bidentate or bridging carboxylate coordination of Ga^3+^ and Sc^3+^ to –COO^−^ groups. For hP4VP, the reduction in intensity and upward shift in the pyridine ring C=N stretching band (~1557 cm^−1^ → ~1611–1638 cm^−1^) and the overall decrease in pyridine ring band intensities across 1650–1100 cm^−1^ confirm direct Ga^3+^/Sc^3+^ coordination through the pyridine nitrogen lone pair, consistent with the formation of M←N dative bonds. As noted in Section 2.5, these FTIR-based coordination assignments should be interpreted alongside the caveat that swelling-induced and hydration-related spectral changes cannot be fully excluded as contributing factors; the mechanistic picture presented here is therefore most robust when supported by the composition-dependent trends (Section 2.8) and the DSC evidence (Section 2.7) considered jointly, rather than by FTIR band shifts in isolation.

It is important to clarify how the outer-sphere (Stage 2) and inner-sphere (Stage 3) pathways are distinguished experimentally in this study, and to what extent they are expected to coexist at equilibrium. Outer-sphere complexes, in which the metal ion retains its hydration shell and associates with the polymer network primarily through long-range electrostatic attraction, are not directly resolved by FTIR, since such complexes do not perturb the vibrational frequencies of the polymer’s functional groups. Inner-sphere complexes, in which one or more water molecules of the metal’s hydration sphere are displaced and a direct coordinate bond forms between the metal center and a carboxylate oxygen or pyridine nitrogen, are the species responsible for the FTIR band shifts documented in Section 2.5 (shifts in ν(C=O), ν(C–O), and ν(C=N)/ring-stretching frequencies). Because FTIR is only sensitive to the inner-sphere population, the spectroscopic evidence presented here should be understood as demonstrating that inner-sphere complexation occurs and is significant at the compositions of maximum sorption, but it cannot quantify what fraction of the total sorbed metal at equilibrium remains in an outer-sphere (electrostatically bound, kinetically labile) state versus an inner-sphere (coordinatively bound, more resistant to acid stripping) state. This distinction is consistent with, and helps explain, the desorption behavior reported in Section 2.2 and Section 2.4: compositions exhibiting the lowest desorption efficiencies (e.g., Sc^3+^ at the 2:4 and 3:3 ratios) are interpreted as having a higher proportion of inner-sphere-bound metal, which is less readily displaced by protonation of the ligand with dilute HNO_3_, whereas compositions with higher desorption efficiencies are interpreted as retaining a larger outer-sphere (electrostatically bound) fraction. Direct experimental separation of the two populations (e.g., by extended-X-ray absorption fine structure (EXAFS) spectroscopy, which can directly resolve first-shell coordination number and metal–ligand bond distances) was outside the scope of the present study and is recommended as a valuable follow-up measurement to quantitatively partition the outer- versus inner-sphere contributions.

Stage 4. Network reorganization and thermodynamic stabilization. The formation of metal–polymer coordination complexes introduces additional crosslinks into the hydrogel networks, partially restricting chain mobility and altering the hydrogen-bonding landscape—as evidenced by the modified DSC heat flow profiles of metal-loaded samples compared to pristine polymers. The TGA data confirm that the overall thermal stability of both hPAA and hP4VP is preserved after metal loading, indicating that the coordination complexes formed are thermodynamically stable under ambient and moderately elevated temperature conditions without inducing backbone degradation.

The overall sorption mechanism may be represented schematically as:

3COO^−^ ⋯M^3+^ ⋯COO− ≡(hPAA, bidentate carboxylate coordination) ≡N:→M^3+^ (hP4VP, dative M←N coordination)

where M^3+^ = Ga^3+^ or Sc^3+^, and the intergel environment enables simultaneous participation of both coordination modes within the same sorbent system. This schematic depicts the inner-sphere coordination modes established in Stage 3; as discussed above, a portion of the total sorbed Ga^3+^ and Sc^3+^ at equilibrium is expected to remain in the outer-sphere, hydrated state depicted in Stage 2, and the relative proportion of each population is not resolved by the present dataset.

The proposed mechanism of the remote interaction effect is supported by Pearson’s Hard and Soft Acid and Base (HSAB) theory [26,27], which states that the hard acid H^+^ and the hard base formed at the hP4VP surface interact to form water molecules, thereby activating and stabilizing the functional groups of both polymer hydrogels within the intergel system. As a result, the concentration of oxonium ions (H_3_O^+^) around hPAA and the concentration of pyridinium species (≡N^+^H) around hP4VP become significantly higher compared to the individual polymers in isolation. This electrochemical gradient lowers the concentration of neutral water molecules in the immediate vicinity of both ion-exchange networks, which in turn enhances the dissociation of counter ions from the ionic groups of the polyelectrolytes and increases the effective density of active coordination sites (–COO^−^ in hPAA and pyridine N in hP4VP). This ultimately leads to the mutual activation of the initial polymer hydrogels and their transition to a highly ionized state, resulting in a significant increase in the sorption properties of the polyacid hPAA and the polybase hP4VP within the intergel system hPAA:hP4VP (X:Y) toward gallium(III) and scandium(III) ions.

This HSAB-based interpretation (Section 2.8) is fully consistent with the mechanistic picture developed above: both Ga^3+^ and Sc^3+^ are hard acids that preferentially engage the hard oxygen donors of hPAA, with Sc^3+^’s marginally larger ionic radius permitting greater participation of the softer pyridine nitrogen donor of hP4VP in its coordination sphere.

### 2.10. Comparative Analysis of Sorption Efficiency

To compare the affinity of the hPAA:hP4VP intergel systems toward gallium(III) and scandium(III), the distribution coefficients K_d_ and the selectivity coefficient β were calculated from the equilibrium concentrations obtained after 48 h of contact. The calculated values are summarized in Table 9 and provide a more rigorous basis for evaluating metal uptake than sorption degree alone, because they reflect not only the amount removed from solution but also the residual equilibrium concentration of each ion.

A clear dependence of sorption efficiency on the composition of the intergel system was observed. For Ga(III), the K_d_ values increased substantially from 694.9 mL·g^−1^ for the individual hPAA hydrogel (6:0) to a maximum of 2726.7 mL·g^−1^ at the equimolar composition 3:3, followed by a moderate decrease at higher hP4VP contents. This trend indicates that the mutual activation of the polyacid and polybase networks under remote interaction conditions markedly enhances gallium binding, with the strongest effect occurring when both components are present in comparable amounts. Even so, the hP4VP-rich systems retained relatively high K_d_ values for Ga(III), confirming that gallium sorption is promoted not only by carboxylate groups of hPAA but also by the cooperative environment created within the intergel pair.

For Sc(III), the distribution coefficients were lower than those for Ga(III) at all investigated compositions, but they also exhibited a pronounced composition dependence. The lowest K_d_ values for Sc(III) were found for the hPAA-dominant systems 5:1 and 4:2, at 217.0 and 247.4 mL·g^−1^, respectively, whereas substantially higher values were obtained for the 3:3 and 2:4 compositions, reaching 1197.8 and 1272.7 mL·g^−1^. These results indicate that scandium uptake is favored in systems containing a larger fraction of hP4VP, which is consistent with the higher sorption degree observed for Sc(III) at 2:4 and 3:3 and with the proposed contribution of pyridine nitrogen atoms to metal coordination.

The selectivity coefficient β = K_d_(Ga)/K_d_(Sc) makes the compositional selectivity of the intergel systems more explicit. In all studied cases, β remained greater than 1, indicating an overall preferential affinity of the hPAA:hP4VP systems toward Ga(III) over Sc(III) under the chosen experimental conditions. The highest selectivity was observed at the 4:2 composition, where β = 6.41, followed by 5:1 (β = 4.52) and 1:5 (β = 4.23). By contrast, the lowest β values were found for the 6:0, 2:4, and 3:3 systems, indicating that although these compositions still favored gallium, their relative affinity for scandium became more pronounced.

The equimolar 3:3 composition deserves special attention. This system exhibited the highest K_d_ for Ga(III) and simultaneously one of the highest K_d_ values for Sc(III), demonstrating that it provides the strongest overall sorption environment among all tested compositions. However, its selectivity coefficient was only moderate (β = 2.28), which suggests that the 3:3 ratio is optimal for maximizing total sorption performance rather than for achieving the strongest discrimination between the two metal ions. In contrast, the 4:2 composition combined a high K_d_ for Ga(III) with a much lower K_d_ for Sc(III), making it the most selective formulation for gallium recovery.

From a mechanistic perspective, these results support the conclusion that the hPAA:hP4VP intergel system can be tuned by varying the polymer ratio not only for high overall sorption capacity but also for the degree of Ga(III)-over-Sc(III) preferential uptake—ranging from near-unity discrimination (β ≈ 1.36 at 6:0) to strongly Ga-selective behavior (β ≈ 6.41 at 4:2)—rather than for reversing the direction of metal preference, which remains Ga(III)-favoring across the entire compositional range studied. The higher affinity for Ga(III) across all compositions is consistent with the stronger contribution of carboxylate-rich environments to gallium binding, whereas the relative increase in Sc(III) uptake in hP4VP-enriched systems suggests a more important role of mixed O/N coordination for scandium. Thus, the comparative K_d_ and β analysis confirms that remote interaction between the hydrogels generates a composition-dependent sorption landscape, allowing targeted adjustment of the intergel system either toward maximum overall uptake or toward enhanced gallium selectivity.

It should be emphasized that the present study was conducted using simplified binary Ga(III)/Sc(III) sulfate model solutions, in the absence of other multivalent cations commonly present in real hydrometallurgical process liquors, such as Al^3+^, Fe^3+^, and rare-earth ions, which typically occur at substantially higher concentrations than Ga(III) and Sc(III) in bauxite-residue and red-mud leachates. Because Al^3+^ and Fe^3+^ are also hard Lewis acids capable of coordinating strongly with carboxylate oxygen donors, their presence is expected to compete for the same –COO^−^ binding sites identified in Section 2.9, potentially reducing the effective Ga(III)/Sc(III) sorption capacity and altering the compositional selectivity trends reported here. Validation of the hPAA:hP4VP intergel system’s performance in the presence of such competing cations, and ultimately in authentic multicomponent leachates, is therefore required before its practical applicability to industrial Ga/Sc recovery can be established, and is identified as a priority for future work.

## 3. Materials and Methods

For this study, two hydrogel-based sorbents were used: (i) polyacrylic acid hydrogel (hPAA), a weakly acidic cation exchanger composed of lightly crosslinked polyacrylate, and (ii) poly-4-vinylpyridine hydrogel (hP4VP), an anion exchanger consisting of cross-linked poly-4-vinylpyridine bearing pyridine nitrogen functional groups.

The following reagents were used: gallium(III) sulfate (purity ≥ 99%, Sigma-Aldrich, Darmstadt, Germany) as a source of Ga^3+^ ions in solution (30 mg/L); scandium(III) sulfate pentahydrate (99.9%, Sigma-Aldrich, Darmstadt, Germany) as a source of Sc^3+^ ions in solution (30 mg/L). Model solutions were prepared in distilled water. A 2 wt.% nitric acid (HNO_3_) solution was used for dilution of samples prior to metal concentration analysis and for desorption experiments. Measurement error did not exceed 5%. All sorption and desorption experiments were performed in triplicate (*n* = 3); reported values are means, with SD not exceeding 5% of the mean.

### 3.1. Preparation of Intergel System

**Figure 19:**
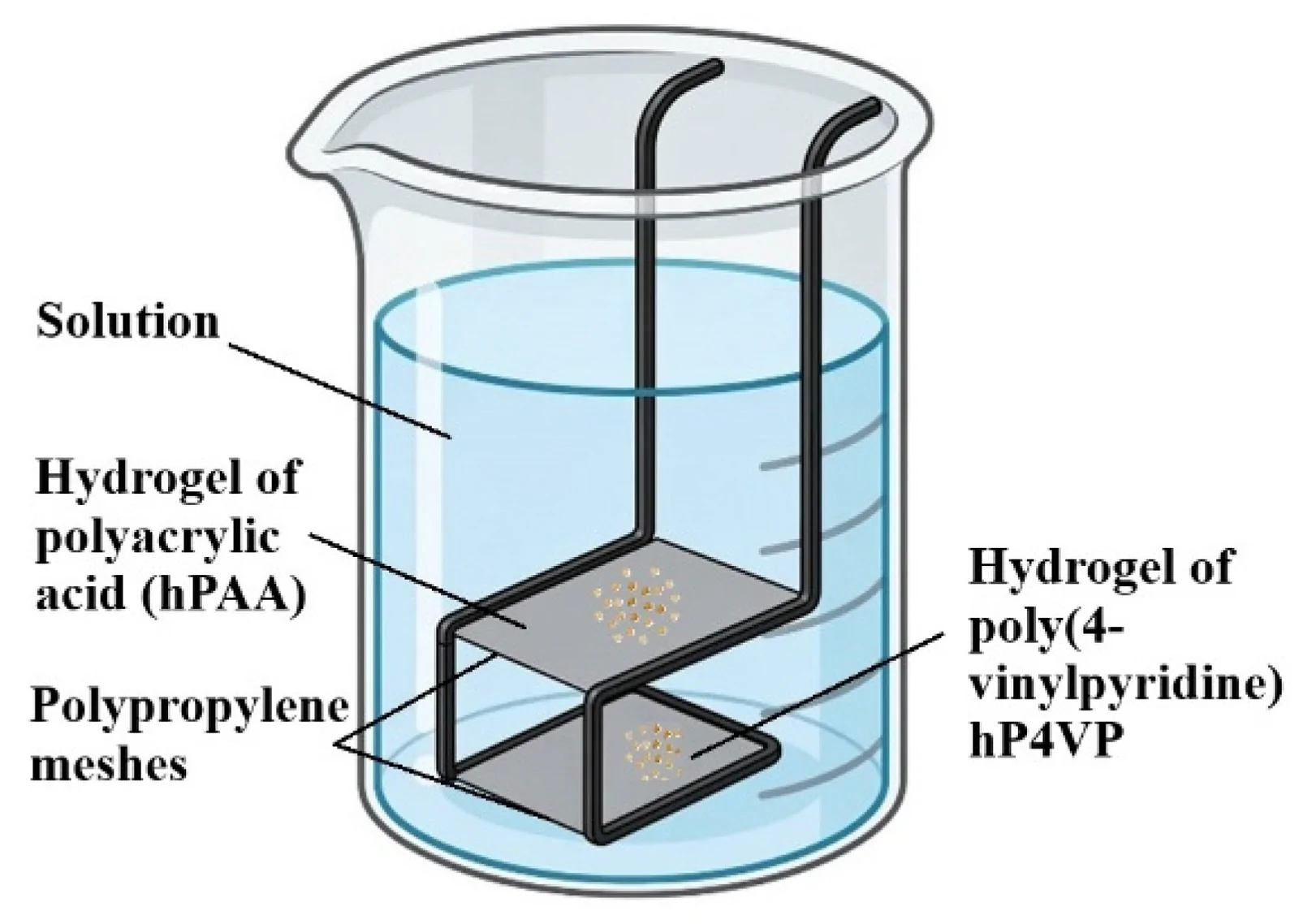
Schematic illustration of the experimental setup for the hPAA:hP4VP intergel system in an aqueous medium: the hydrogel of polyacrylic acid (hPAA) and the hydrogel of poly(4-vinylpyridine) (hP4VP) are placed in separate polypropylene mesh compartments and simultaneously immersed in the binary mixed Ga^3+^/Sc^3+^ aqueous solution, enabling remote (non-contact) interpolymer interaction without direct physical mixing of the two hydrogel networks.

The sorption experiments were conducted according to the setup illustrated in Figure 19. The two polymer hydrogels were physically separated by individual polypropylene mesh enclosures and immersed simultaneously in the binary metal ion solution, ensuring that the mutual activation effect of the interpolymer system arose exclusively from remote interpolymer interaction through the aqueous medium rather than from direct polymer–polymer contact. This experimental design, first proposed by Jumadilov et al. [10], is a defining characteristic of intergel systems and is essential for isolating the synergistic sorption enhancement attributable to the mutual activation effect from any mechanical mixing effects.

**Scheme 1:**
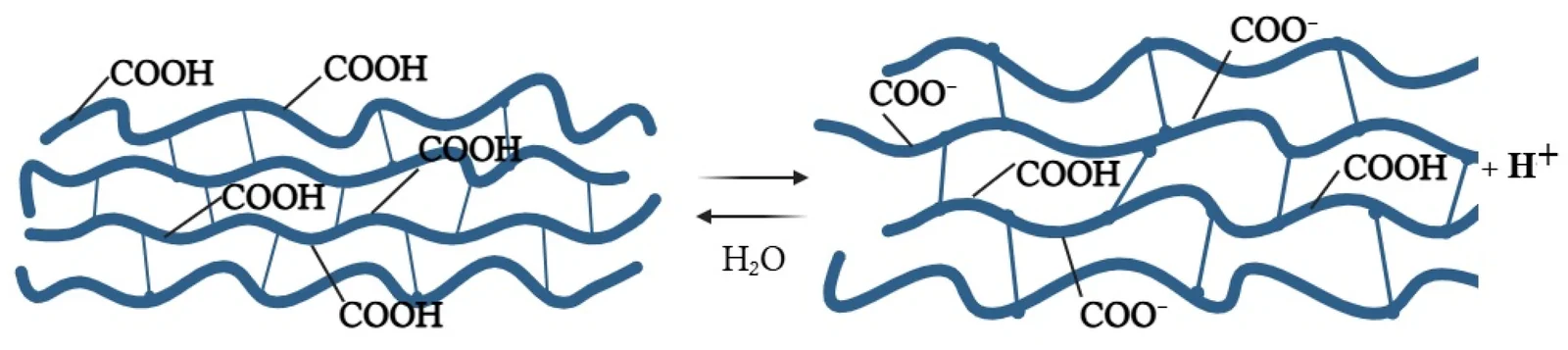
hPAA dissociates in an aqueous solution according to Scheme 1.

**Scheme 2:**
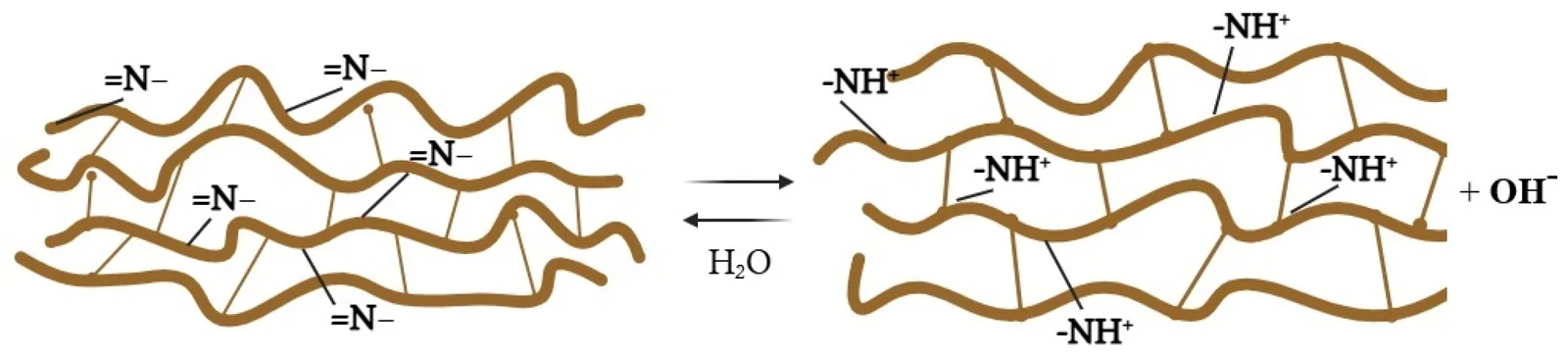
hP4VP dissociates in an aqueous solution according to Scheme 2.

For sorption, the polypropylene meshes with swollen hPAA and hP4VP were placed in a glass with distilled water at about 1–2 cm apart (Figure 1). As is known [12,13,14], the acid-base properties of the ion-exchange polymer hydrogels hPAA (H^+^ form) and hP4VP (free base form) are associated with their dissociation with the release of H^+^ and the acceptance of H^+^ ions by the pyridine nitrogen, respectively. Free H^+^ ions (Scheme 1) leave the carboxylic functional groups of hPAA without counterions, stabilized by intramolecular hydrogen-bonding interactions, while the pyridine nitrogen atoms of hP4VP accept these protons (Scheme 2), forming pyridinium species (≡N^+^H) and generating hydroxyl ions in the surrounding aqueous medium. As a result, the electrochemical and conformational changes in both macromolecular networks lead to an increased sorption capacity of the intergel system hPAA:hP4VP (X:Y) toward gallium(III) and scandium(III) ions in comparison with the individual hydrogels hPAA (6:0) and hP4VP (0:6).

Figure 19 illustrates the schematic setup for the sorption process using the hPAA:hP4VP intergel system (X:Y) in aqueous medium. The schematic depicts a glass beaker containing the binary mixed Ga^3+^/Sc^3+^ aqueous solution, into which two physically separated polymer hydrogel samples are simultaneously immersed. The hydrogel of polyacrylic acid (hPAA) and the hydrogel of poly(4-vinylpyridine) (hP4VP) are each enclosed within individual polypropylene mesh compartments, which prevent direct physical contact between the two polymer networks while permitting free diffusion of ions and solvent molecules through the mesh. The polypropylene mesh thus serves as a permeable physical barrier that enables the remote (non-contact) interaction between hPAA and hP4VP—the defining feature of the intergel system—while ensuring that sorption data reflect the properties of each polymer individually within the shared aqueous environment.

All sorption and desorption experiments were conducted at ambient temperature (25 ± 1 °C) under continuous magnetic stirring at 300–400 rpm, unless otherwise stated. The mixed Ga(III)/Sc(III) sulfate solutions were prepared in distilled water with initial metal concentrations of 30 mg·L^−1^ each and an initial pH of 2.5 ± 0.1, measured with a 780 pH meter (Metrohm, Switzerland). The target pH was achieved by adding dilute H_2_SO_4_ (0.1 mol·L^−1^) dropwise to the as-prepared Ga(III)/Sc(III) sulfate solutions, with pH monitored continuously until the target value was reached and stable for ≥5 min. The total solution volume in each experiment was V_0_ (e.g., 100 mL), and the mass of each dry hydrogel sample was m (e.g., 0.1 g), corresponding to a solid-to-liquid ratio of m/V_0_. These conditions were kept constant across all intergel compositions to allow direct comparison of sorption and desorption performance.

The solution pH is expected to be a critical parameter governing the degree of ionization of both hPAA (pK_a_ ≈ 4.50–4.75) and hP4VP (pK_b_ of the pyridine nitrogen, typically pK_a_(pyridinium) ≈ 4.90–5.20 for poly(4-vinylpyridine)). At the acidic pH imposed by dilute H_2_SO_4_ in the present experiments, hPAA carboxyl groups are only partially ionized while hP4VP pyridine nitrogens are substantially protonated; this initial ionization state directly determines the density of –COO^−^ and pyridinium/free-base nitrogen sites available for mutual activation and metal coordination. Because the degree of ionization of both polyelectrolytes is pH-dependent, variations in the initial pH would be expected to shift the balance between carboxylate-driven (hard, oxygen-donor) and pyridine-driven (softer, nitrogen-donor) coordination environments, and hence to modulate the Ga(III)/Sc(III) selectivity reported in Section 2.8. A systematic investigation of sorption and selectivity as a function of initial solution pH was outside the scope of the present study, which focused on composition (hPAA:hP4VP ratio) as the primary tunable variable; this pH dependence is identified as an important direction for future work (see Section 2.10 and Section 4).

There are two main dissociation equilibria of the polymer hydrogels that occur in an aqueous medium and constitute the mechanistic basis of the mutual activation effect in the hPAA:hP4VP intergel system.

As illustrated in Scheme 1 (step 1), hPAA undergoes partial deprotonation of its carboxylic acid groups (–COOH) in water according to the equilibrium (1):

≡COOH + H_2_O ⇌ ≡COO− + H^+^, (1)

This process generates negatively charged carboxylate anions (–COO^−^) distributed along the polyacrylate backbone, which serve as the primary coordination sites for Ga^3+^ and Sc^3+^ ions, together with free protons (H^+^) released into the surrounding aqueous medium. The equilibrium is shifted toward the deprotonated form at pH values above the pKa of polyacrylic acid (~4.50–4.75).

As illustrated in Scheme 2 (step 2), hP4VP undergoes protonation of its pyridine ring nitrogen atoms (=N–) by water molecules according to the equilibrium (2):

≡N: + H_2_O ⇌ ≡NH+ + OH−, (2)

This process generates positively charged pyridinium species (–N^+^) along the poly(4-vinylpyridine) backbone, together with hydroxide ions (OH^−^) released into the surrounding medium. Within the intergel system, the H^+^ ions released by hPAA (step 1) and the OH^−^ ions generated by hP4VP (step 2) combine to form water molecules, thereby shifting both equilibria toward higher degrees of ionization—the fundamental driving force of the mutual activation effect. This enhanced ionization leads to a significantly increased density of active coordination sites (–COO^−^ in hPAA and = N– in hP4VP) compared to either individual hydrogel in isolation, which directly accounts for the superior sorption performance of the intergel system toward Ga^3+^ and Sc^3+^ ions relative to the individual polymer components.

It should be noted that, although no exogenous pH adjustment (beyond the initial pH-2.5 conditioning described above) was applied during the sorption stage itself, the hPAA:hP4VP intergel system inherently and locally modifies the acid–base environment of the solution through the H^+^ release from hPAA and OH^−^ release from hP4VP described in Equations (1) and (2); this local perturbation of solution chemistry is, in fact, the proposed mechanistic basis of the mutual activation effect (Section 2.9) rather than an unintended artifact. Any statement elsewhere in this manuscript describing sorption as proceeding ‘without modification of solution chemistry’ refers specifically to the absence of deliberate, external chemical intervention (e.g., titrant addition or buffering) during the sorption stage, and should not be read as implying the intergel system itself has no effect on local pH or ionic equilibria.

### 3.2. Equipment

Specific electrical conductivity was measured with a benchtop conductometer S230-USP/EP (Mettler Toledo, Columbus, OH, USA), which was used to assess the dissociation behavior of the polyelectrolytes and the ionic charge transport in scandium-containing solutions. The acid–base properties of the solutions were evaluated using a 780-pH meter (Metrohm, Herisau, Switzerland) to determine the hydrogen ion concentration. Sample masses were determined on an analytical balance AND HR-100 AZG (A&D Company, Ltd., Tokyo, Japan). The residual scandium content in aqueous samples after treatment was quantified by inductively coupled plasma optical emission spectroscopy using an iCAP PRO XP spec- 881 trometer (Thermo Fisher Scientific, Loughborough, Leicestershire, UK) operating in the wavelength range of 165–782 nm. Functional groups and coordination interactions within the intergel system were analyzed using Fourier transform infrared (FTIR) spectroscopy (Thermo Fisher Scientific, Madison, WI, USA). Spectra were acquired in transmission mode over the 500–4000 cm^−1^ region using a Nicolet 5700 spectrometer (Thermo Fisher Scientific, Madison, WI, USA). Thermogravimetric measurements were performed on a synchronous thermogravimetric analyzer SKZ1053 (SKZ 887 Industrial Co., Ltd., Dongguan, China), which was used to evaluate the thermal stability and mass loss behavior of the polymer samples. Thermal behavior, including stability and decomposition of PAA and P4VP polymers before and after sorption, was evaluated using thermogravimetric analysis (TG 209 F3, NETZSCH, Selb, Germany). Measurements were carried out in the temperature range of 35–800 °C using sample masses between 3.38 and 4.72 mg. Both mass loss and associated thermal events were monitored to assess structural changes in the sorbent materials.

### 3.3. Experimental Procedures

In order to determine the residual concentrations of gallium(III) and scandium(III) ions in the aqueous samples collected after sorption and desorption experiments, inductively coupled plasma optical emission spectrometry (ICP-OES) was performed using a PerkinElmer Optima 8300 DV spectrophotometer (PerkinElmer, Waltham, MA, USA). In the first step, a series of standard 900 solutions of Ga^3+^ and Sc^3+^ of known concentrations ranging from 0 to 100 mg/L were prepared by serial dilution of certified single-element standard solutions in deionized water. The selected sample solutions were diluted with deionized water to bring the analyte concentrations within the linear range of the calibration curve prior to measurement. Calibration curves were constructed by measuring the emission intensity of each standard solution at the characteristic analytical wavelengths of gallium (294.364 nm) and scandium 906 (361.383 nm), respectively. The sample solutions were then measured under identical instrumental conditions, with the spectrophotometer set to scan the wavelength range from 167 to 800 nm.

### 3.4. Data Treatment

The degree of sorption (η, %) was calculated according to Equation (3):

(3) $$\eta =\frac{C_{0}-C_{eq}}{C_{0}}\times 100\%,$$

where C_0_ (mg·L^−1^) is the initial concentration of metal ions (Ga^3+^ or Sc^3+^) in solution, and C_eq_ (mg·L^−1^) is its equilibrium (residual) concentration in solution after the specified contact time, as determined by ICP-OES.

The degree of desorption (R, %) was calculated according to Equation (4):

(4) $$R=\frac{C_{des}\cdot V_{des}}{m_{sorbed}}\times 100\%$$

where C_des_–concentration of metal ions in the desorbate solution, mg·L^−1^; V_des_ (L) is the total volume of the desorbent solution (2 wt.% HNO_3_) used in the desorption step; and m_sorbed_ (mg) is the total mass of metal ions taken up by the sorbent during the preceding sorption stage, calculated as: m_sorbed_ = (C_0_ − C_eq_)·V_0_, where C_0_ (mg·L^−1^) is the initial metal-ion concentration in the sorption solution, C_eq_ (mg·L^−1^) is its equilibrium concentration after the sorption contact time (48 h), and V_0_ (L) is the volume of the sorption solution.

The distribution coefficients K_d_ (mL·g^−1^) for Ga(III) and Sc(III) were determined from equilibrium concentration data according to Equation (5):

(5) $$K_{d}=\frac{C_{i}-C_{eq}}{C_{eq}}\times \frac{V}{m},$$

where C_i_ and C_eq_ (mg·L^−1^) denote the initial and equilibrium concentrations of the metal ions, respectively, V (mL) is the solution volume, and m (g) is the mass of the dry sorbent.

The selectivity coefficient β was calculated using Equation (6):

(6) $$\beta =\frac{K_{d}(Ga)}{K_{d}(Sc)},$$

These parameters were employed to quantitatively assess the selectivity and affinity of the PAA/P4VP intergel system toward Ga(III) and Sc(III) ions in solution. By comparing the distribution coefficients and their ratio, it becomes possible to determine the extent to which one metal ion is preferentially adsorbed over the other under identical experimental conditions. This analysis provides insight into the competitive sorption behavior, the influence of polymer functional groups on metal binding, and the overall efficiency of the intergel system in selectively recovering Ga(III) or Sc(III) from mixed-metal solutions.

### 3.5. Langmuir and Freundlich Isotherm Models

The equilibrium sorption behavior of Ga(III) and Sc(III) on the hPAA:hP4VP intergel system was further analyzed using the Langmuir adsorption model. The Langmuir equation assumes monolayer adsorption onto a homogeneous surface with a finite number of equivalent sorption sites and can be written as the following linearized Langmuir Equation (7):

(7) $$\frac{C_{e}}{q_{e}}=\frac{1}{Q_{max}K_{L}}+\frac{C_{e}}{Q_{max}}$$

where Q_m_ is the maximum adsorption capacity (anions mg/g of resin), K_L_ is the Langmuir equilibrium constant (L/mg), and C_e_ is the equilibrium concentration of the adsorbate (mg/L).

In the present work, equilibrium data for Ga(III) and Sc(III) were obtained from binary sulfate solutions after 48 h of contact between the mixed metal solution and the hPAA:hP4VP intergel, with initial concentrations C_0_(Ga) = 196.97 mg·L^−1^ and C_0_(Sc) = 97.596 mg·L^−1^. For a series of experiments conducted at different combinations of metal loading and sorbent mass, the residual equilibrium concentrations C_e_ of Ga(III) and Sc(III) spanned the ranges 131.09 ÷ 7.43 mg·L^−1^ and 92.690 ÷ 3.788 mg·L^−1^, respectively.

Preliminary Langmuir plots constructed using these relative q_e_ values exhibited approximately linear behavior for Ga(III) over the studied concentration range, with correlation coefficients R^2^ = 0.94803, whereas the Sc(III) data showed more pronounced deviations from linearity (R^2^ ≈ 0.84). These results suggest that Ga(III) sorption on the intergel system is more consistent with a Langmuir-type monolayer mechanism than Sc(III) sorption, which appears to be influenced to a greater extent by heterogeneity and/or competitive effects in the binary system.

Qualitatively, the equilibrium data clearly indicate that the intergel system “hPAA:hP4VP” exhibits a higher relative uptake of Ga(III) than Sc(III) under identical initial concentrations and contact conditions, consistent with the higher sorption degrees observed at the optimal compositions (e.g., ~79% for Ga(III) at 3:3 versus ~60% for Sc(III) at 2:4). This trend can be rationalized by the strong interaction of Ga(III) with deprotonated carboxylate sites in hPAA and additional coordination at pyridine nitrogens in hP4VP, whereas Sc(III) sorption is governed to a greater extent by pyridine coordination and is more sensitive to the hP4VP content. The apparent Langmuir-like behavior of Ga(III) suggests that the intergel system approaches a finite saturation capacity for Ga(III) under the studied conditions.

In addition to the Langmuir model, the Freundlich isotherm was applied to evaluate the possible contribution of multilayer or heterogeneous-site sorption behavior, since the Langmuir model assumes a homogeneous, single-site monolayer mechanism that may not fully describe the more scattered equilibrium data obtained for Sc(III). The Freundlich equation is expressed as Equation (8):

(8) $$q_{e}=K_{F}\cdot C_{e}^{\frac{1}{n}}$$

which, in linearized (logarithmic) form suitable for regression analysis, becomes Equation (9):

(9) $${log}_{q_{e}}={logK}_{F}+\frac{1}{n}\cdot logC_{e}$$

where q_e_ (mg·g^−1^) is the equilibrium sorption capacity of the sorbent, C_e_ (mg·L^−1^) is the equilibrium concentration of the metal ion remaining in solution, K_F_ ((mg·g^−1^)·(L·mg^−1^)^1/n^) is the Freundlich affinity constant reflecting the relative sorption capacity of the sorbent, and n (dimensionless) is the Freundlich heterogeneity exponent; values of 1/n between 0 and 1 indicate favorable, increasingly heterogeneous sorption, whereas 1/n > 1 is generally associated with cooperative sorption behavior. A linear plot of log q_e_ versus log C_e_ yields K_F_ from the intercept and 1/n from the slope.

**Figure 20:**
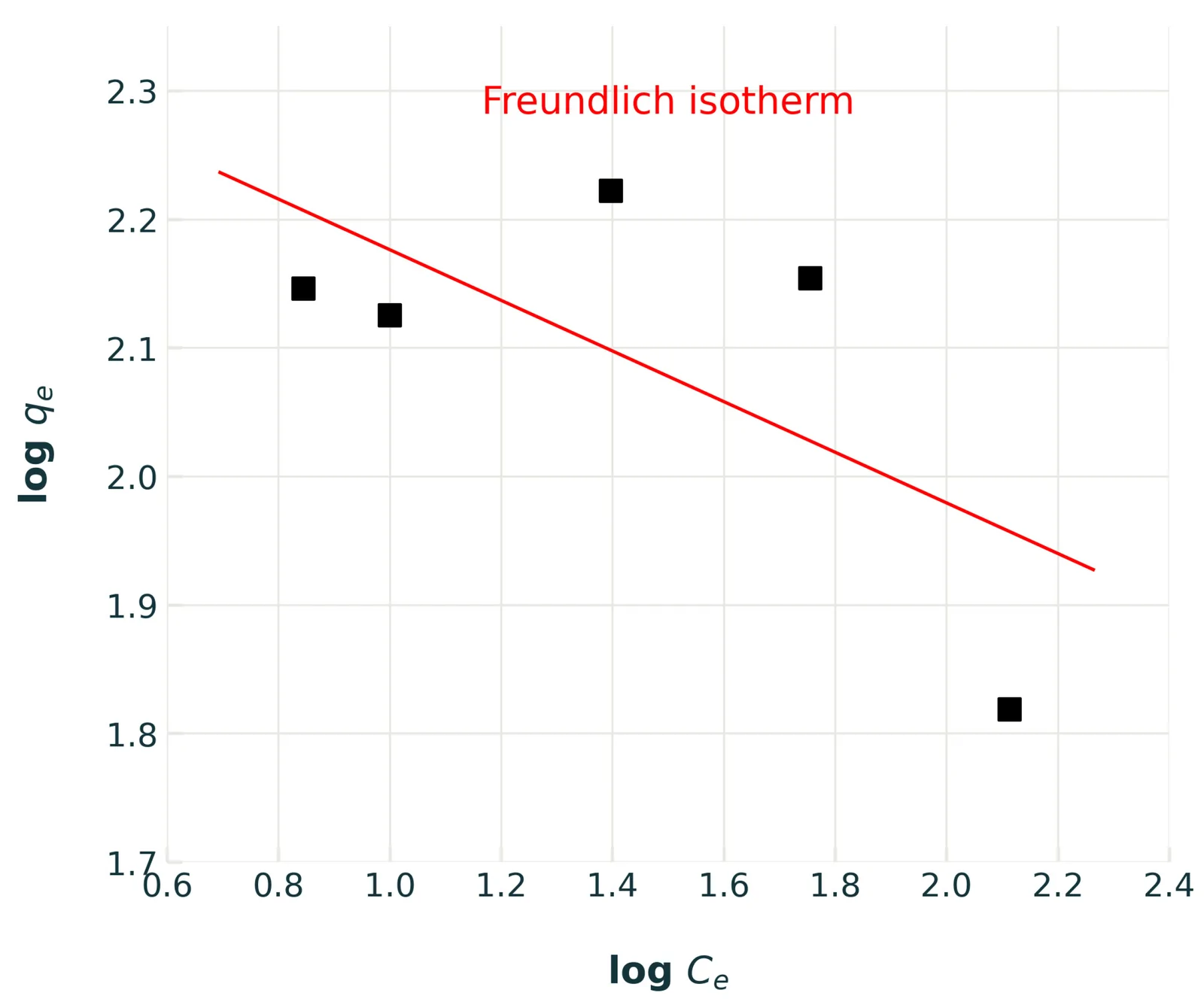
Freundlich isothermal model for the intergel system “hPAA:hP4VP” (3:3), Ga(III). The black squares (■) represent the experimental equilibrium data points (C_e_ = 7, 10, 25, 57, and 130 mg·L^−1^), and the solid red line corresponds to the linear regression of the linearized Freundlich equation, log qe = log K_F_ + (1/n)·log Ce. The fitted parameters (K_F_, n, R^2^) are given in the inset.

Qualitatively, the equilibrium data plotted in Figure 20 do not follow the rising, approximately linear trend expected of a valid Freundlich isotherm. The linear regression of log q_e_ against log C_e_ yields K_F_ = 236.1 (mg·g^−1^)(L·mg^−1^)^1/n^ and n = −5.08 (1/n = −0.197), with R^2^ = 0.429. Because the Freundlich exponent n is required to be positive under the physical assumptions of the model—with 1/n values between 0 and 1 denoting favorable, increasingly heterogeneous sorption—a negative n has no defined physical interpretation, and this fit cannot be reported as a valid Freundlich parameterization of Ga(III) sorption on the hPAA:hP4VP (3:3) system.

To further test whether the Ga(III) equilibrium data are more consistent with a homogeneous, single-site monolayer mechanism (Langmuir) or a heterogeneous, multi-site sorption mechanism (Freundlich), the same equilibrium dataset used to construct the Langmuir plot was fitted to the linearized Freundlich equation (Equation (9)). The resulting Freundlich plot for the hPAA:hP4VP (3:3) system is shown in Figure 20. The five experimental points, spanning Ce = 7–130 mg·L^−1^ and qe = 65.8–166.7 mg·g^−1^, do not fall along a line with a physically interpretable positive slope: the linear regression of log q_e_ against log Ce yields an apparent heterogeneity exponent of n = −5.08 (1/n = −0.197) and K_F_ = 236.1, with R^2^ = 0.429. Because the Freundlich exponent n is required to be positive under the physical assumptions of the model—values of 1/n between 0 and 1 denote favorable, increasingly heterogeneous sorption, whereas negative values have no defined physical interpretation—this fit cannot be reported as a valid Freundlich parameterization of the Ga(III) sorption equilibrium.

**Figure 21:**
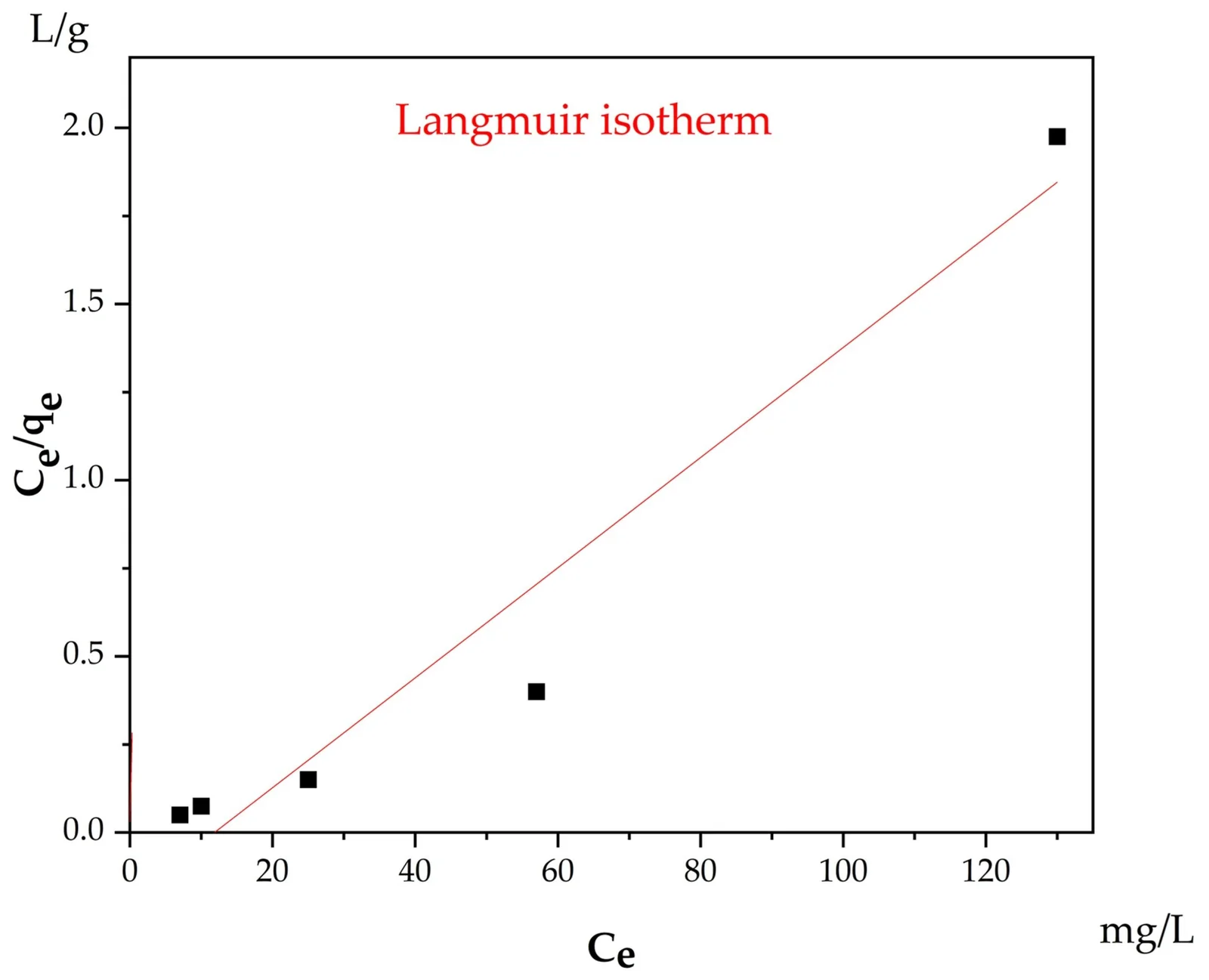
Langmuir isothermal model for the intergel system “hPAA:hP4VP” (3:3) Ga(III). The black squares (■) represent the experimental data points, and the solid line corresponds to the Langmuir isotherm fit.

Inspection of the underlying data clarifies the origin of this result: the equilibrium uptake qe does not increase monotonically with the residual equilibrium concentration Ce across the five conditions tested, rising from 140.0 mg·g^−1^ at Ce = 7 mg·L^−1^ to a maximum of 166.7 mg·g^−1^ at Ce = 25 mg·L^−1^, before decreasing to 142.5 and 65.8 mg·g^−1^ at Ce = 57 and 130 mg·L^−1^, respectively. This same non-monotonic trend is reflected in the Langmuir analysis: although the linearized Langmuir plot (Figure 21) returns an outwardly acceptable correlation coefficient (R^2^ = 0.948), back-calculation of the Langmuir affinity constant from this fit yields KL = −0.084 L·mg^−1^, which is likewise physically inadmissible, since K_L_ must be positive. Taken together, these findings indicate that a linear R^2^ value alone is not sufficient evidence of a physically valid isotherm fit, and that the present five-point equilibrium dataset—collected across a limited range of initial Ga(III) loadings and/or sorbent doses—does not yet permit an unambiguous mechanistic assignment of Ga(III) sorption to either a homogeneous monolayer or a heterogeneous multi-site model. We report this outcome transparently, rather than presenting either set of fitted isotherm constants (Q_max_, K_L_, K_F_, n) as definitive, and identify the collection of a larger and more closely spaced equilibrium dataset—ideally spanning a wider concentration range with additional intermediate points—as a priority for future quantitative validation of the Ga(III) sorption mechanism proposed in Section 2.9.

## 4. Conclusions

This study systematically investigated the sorption of gallium(III) and scandium(III)—two critical, strategically important metals—by the hPAA:hP4VP intergel system across seven mass ratios (6:0 to 0:6) in binary sulfate solutions. The hPAA:hP4VP system exhibits a pronounced mutual activation effect: combining hPAA and hP4VP under remote-contact conditions increases the density of active coordination sites (–COO^−^ and pyridine N) relative to either individual hydrogel, yielding higher sorption of both Ga^3+^ and Sc^3+^ than the corresponding single-component controls. [AUTHORS: insert the recalculated maximum sorption degrees for Ga^3+^ and Sc^3+^, and their corresponding optimal ratios, once Table 2 and Table 6 have been recomputed per Reviewer 3, Comments 1–2] at 48 h contact time. This composition-dependent divergence reflects differing coordination preferences —Ga^3+^ favoring oxygen-donor carboxylate-rich environments and Sc^3+^ tolerating a broader mixed O/N environment—providing a basis for composition-tunable Ga/Sc processing using a single sorbent platform.

Equilibrium sorption data for Ga(III) were further analyzed using both the Langmuir and Freundlich isotherm models. While the linearized Langmuir plot returned an outwardly acceptable correlation coefficient (R^2^ ≈ 0.95), both the back-calculated Langmuir affinity constant and the Freundlich heterogeneity exponent obtained from the same equilibrium dataset were physically inadmissible (negative), reflecting a non-monotonic relationship between equilibrium uptake and residual concentration across the five conditions tested. This finding indicates that the present isotherm dataset is too limited to support a definitive mechanistic assignment of Ga(III) sorption to either a homogeneous monolayer or a heterogeneous multi-site model, and expansion of the equilibrium dataset is identified as a priority for future quantitative validation of the proposed sorption mechanism. FTIR spectroscopy indicated that both metals form inner-sphere complexes—bidentate carboxylate coordination in hPAA and dative M←N coordination in hP4VP—although alternative contributions from polymer swelling, hydrogen-bonding reorganization, and hydration-shell changes cannot be fully excluded on the basis of FTIR alone (see Section 2.5, revised discussion). TGA/DSC analysis confirmed that both polymers retain structural integrity up to approximately 320–340 °C after metal loading, with the onset of the main hP4VP decomposition event at ~350 °C; this establishes adequate thermal stability under the tested conditions but does not, by itself, demonstrate industrial applicability, which additionally requires validation in the presence of competing multivalent cations (e.g., Al^3+^, Fe^3^) and in authentic process liquors; moreover, because Ga(III)/Sc(III) sorption and desorption are expected to occur near ambient temperature in practice, the demonstrated thermal stability window (320–340 °C) substantially exceeds the temperatures relevant to routine process operation and should be interpreted as establishing material robustness under extreme conditions rather than as a direct proxy for process performance.

Overall, the results demonstrate that Ga(III) is preferentially taken up over Sc(III) across all seven compositions studied (β > 1 in every case), indicating a consistent Ga(III) preference rather than a switch in target-metal selectivity; the composition dependence of the magnitude of this preference (and of the total uptake) nonetheless offers a practical route to tune the relative recovery of the two metals. The findings support the hPAA:hP4VP intergel system as a promising, composition-tunable platform for the recovery of Ga(III) and, to a lesser extent, Sc(III) from simplified sulfate media, while highlighting initial pH control, competing-ion tolerance, and validation in real hydrometallurgical liquors as necessary next steps toward practical implementation.

## Figures and Tables

**Table 1:** Equilibrium concentration of Ga^3+^ ions in a mixture of solutions after the sorption process using the hPAA:hP4VP intergel system, depending on the polymer ratio and contact time.

hPAA:hP4VP (X:Y)							
Molar ratio (X:Y)	6:0	5:1	4:2	3:3	2:4	1:5	0:6
Contact time, h	Ga^3+^ concentration, mg/L						
0.5	25.65	26.45	26.10	27.65	27.70	27.20	28.85
1	28.05	23.95	25.50	24.10	26.65	25.20	25.60
2	23.95	23.30	24.25	22.85	22.35	22.55	23.05
4	21.35	20.50	19.75	21.3	20.05	19.30	20.10
6	19.45	17.95	20.65	19.00	17.45	18.75	17.40
24	19.25	15.75	13.40	11.80	11.70	12.40	12.45
48	17.70	15.15	11.60	8.05	9.85	11.40	11.70

**Table 2:** Degree of sorption (%) of galli um(III) ions by the intergel system hPAA:hP4VP. In mixed solution after the sorption process, as a function of polymer mass ratio and contact time.

hPAA:hP4VP (X:Y)							
Molar ratio (X:Y)	6:0	5:1	4:2	3:3	2:4	1:5	0:6
Contact time, h	Ga^3+^ concentration, mg/L						
0.5	14.50	11.83	13.00	7.83	7.67	9.33	3.83
1	16.50	20.17	15.00	19.67	11.17	16.00	14.67
2	20.17	22.33	19.17	23.83	25.50	24.83	23.17
4	28.83	31.67	31.17	29.00	33.17	35.67	33.00
6	35.17	40.17	34.17	36.67	41.83	37.50	42.00
24	35.83	47.50	55.33	60.67	61.00	58.67	58.50
48	41.00	49.50	61.33	73.17	67.17	62.00	61.00

**Table 3:** Residual concentration of gallium(III) ions in mixed solution after the desorption process as a function of polymer mass ratio (contact time: 48 h, desorbent: 2 wt.% HNO_3_).

hPAA:hP4VP (X:Y)							
Molar ratio (X:Y)	6:0	5:1	4:2	3:3	2:4	1:5	0:6
Contact time	Ga^3+^ concentration, mg/L						
48 h	13.60	10.55	13.95	14.70	12.20	10.80	10.15

**Table 4:** Degree of desorption (%) of gallium(III) ions by the intergel system hPAA:hP4VP in mixed solution after the desorption process, as a function of polymer mass ratio (contact time: 48 h, desorbent: 2 wt.% HNO_3_).

hPAA:hP4VP (X:Y)							
Molar ratio (X:Y)	6:0	5:1	4:2	3:3	2:4	1:5	0:6
Time, h	Ga^3+^ concentration, mg/L						
48	55.41	71.04	75.82	66.97	60.55	58.06	55.46

**Table 5:** Residual concentration of Sc^3+^ ions in the intergel system hPAA:hP4VP in mixed solution after the sorption process, as a function of polymer mass ratio and contact time.

hPAA:hP4VP (X:Y)							
Molar ratio (X:Y)	6:0	5:1	4:2	3:3	2:4	1:5	0:6
Contact time, h	Sc^3+^ concentration, mg/L						
0.5	32.10	34.25	33.75	34.25	33.65	33.90	33.70
1	36.50	32.30	34.50	32.60	34.60	33.60	32.05
2	33.00	34.20	34.85	32.20	32.25	32.65	31.25
4	30.85	33.15	31.65	32.50	29.90	31.00	30.45
6	29.00	30.30	34.85	29.30	26.8	32.10	28.45
24	24.70	27.95	27.65	19.00	17.25	24.55	23.20
48	19.85	24.65	24.05	13.65	13.20	21.65	18.80

Note. Values are expressed as residual Sc^3+^ concentration (mg/L). Initial concentration C_0_ = 30 mg/L. Metal concentrations were determined individually by ICP-OES (iCAP PRO XP, Thermo Scientific, USA) despite the binary nature of the solution.

**Table 6:** Calculated degrees of Sc^3+^ sorption (η) derived from the equilibrium concentration data obtained by ICP-OES for the mixed gallium–scandium solution.

hPAA:hP4VP (X:Y)							
Molar ratio (X:Y)	6:0	5:1	4:2	3:3	2:4	1:5	0:6
Time, h	Sc^3+^ concentration, mg/L						
0.5	2.58	1.04	1.42	0.94	1.12	0.88	1.27
1	1.15	1.97	1.70	1.06	2.01	0.97	2.73
2	1.77	3.79	2.76	2.27	3.12	2.91	5.16
4	6.37	6.61	3.94	2.36	9.25	5.91	7.58
6	11.98	6.83	5.76	11.07	18.66	2.57	13.65
24	17.67	8.04	7.83	36.67	42.50	18.17	22.67
48	33.83	17.83	19.83	54.50	56.00	27.83	37.33

Note. Values are expressed as sorption degree η (%) = [(C_0_ − C_e_q)/C_0_] × 100%, where C_0_ = 30 mg/L.

**Table 7:** Residual concentration of scandium(III) ions in mixed solution after the desorption process, as a function of polymer mass ratio (contact time: 48 h, desorbent: 2 wt.% HNO_3_).

hPAA:hP4VP (X:Y)							
Molar ratio (X:Y)	6:0	5:1	4:2	3:3	2:4	1:5	0:6
Time, h	Sc^3+^ concentration, mg/L						
48	16.55	11.65	13.20	21.40	22.00	14.35	17.30

Note. Values are expressed as residual Sc^3+^ concentration (mg/L) in the desorbing 2 wt.% HNO_3_ solution after 48 h of contact. Initial concentration C_0_ = 30 mg/L.

**Table 8:** Degree of desorption (%) of scandium(III) ions by the intergel system hPAA:hP4VP in mixed solution after the desorption process, as a function of polymer mass ratio (contact time: 48 h, desorbent: 2 wt.% HNO_3_).

hPAA:hP4VP (X:Y)							
Molar ratio (X:Y)	6:0	5:1	4:2	3:3	2:4	1:5	0:6
Time, h	Sc^3+^ concentration, mg/L						
48	44.64	61.03	55.85	28.43	26.42	52.00	42.14

Note. Values are expressed as desorption degree (%) = (C_des_ × V_des_/m_sorbed_) × 100%, where m_sorbed_ = (C_0_ − C_eq_)·V_0_ (Equation (4)), using C_0_ = 30 mg/L and V_0_ = V_des_ = 100 mL.

**Table 9:** Distribution and Selectivity Coefficients for Ga(III) and Sc(III) on hPAA:hP4VP Intergel Systems (48 h, Binary Sulfate Solution).

hPAA:hP4VP	C_eq,Ga_ (mg·L^−1^)	C_eq,Sc_ (mg·L^−1^)	K_d(Ga)_ (mL·g^−1^)	K_d(Sc)_ (mL·g^−1^)	β (K_d_,_Ga_)/K_d,Sc_)	Selectivity
6:0	17.70	19.85	694.9	511.3	1.36	Ga-selective
5:1	15.15	24.65	980.2	217.0	4.52	Ga-selective
4:2	11.60	24.05	1586.2	247.4	6.41	Ga-selective
3:3	8.05	13.65	2726.7	1197.8	2.28	Ga-selective
2:4	9.85	13.20	2045.7	1272.7	1.61	Ga-selective
1:5	11.40	21.65	1631.6	385,7	4.23	Ga-selective
0:6	11.70	18.80	1564.1	595.7	2.63	Ga-selective

## Data Availability

The data presented in this study are available upon request from the corresponding author.

## Abbreviations

The following abbreviations are used in this manuscript:

hPAA	Hydrogel of Poly(acrylic acid)
hP4VP	Hydrogel of Poly(4-vinylpyridine)
HSAB	Hard and Soft Acids and Bases (Pearson HSAB theory)
